# ASTEROID: A New Clinical Stereotest on an Autostereo 3D Tablet

**DOI:** 10.1167/tvst.8.1.25

**Published:** 2019-02-28

**Authors:** Kathleen Vancleef, Ignacio Serrano-Pedraza, Craig Sharp, Gareth Slack, Carla Black, Therese Casanova, Jess Hugill, Sheima Rafiq, James Burridge, Vito Puyat, Josee Ewane Enongue, Henry Gale, Hannah Akotei, Zoe Collier, Helen Haggerty, Kathryn Smart, Christine Powell, Kate Taylor, Michael P. Clarke, Graham Morgan, Jenny C. A. Read

**Affiliations:** 1 Institute of Neuroscience, Newcastle University, Framlington Place, Newcastle upon Tyne, UK; 2 Facultad de Psicología, Universidad Complutense de Madrid, Campus de Somosaguas, Madrid, Spain; 3 School of Computing, Newcastle University, 1 Science Square, Newcastle upon Tyne, UK; 4 Newcastle Eye Centre, Royal Victoria Infirmary, Newcastle upon Tyne Hospitals NHS Trust, Queen Victoria Road, Newcastle upon Tyne, UK

**Keywords:** stereoacuity, binocular vision, psychophysics, depth perception, stereopsis

## Abstract

**Purpose:**

To describe a new stereotest in the form of a game on an autostereoscopic tablet computer designed to be suitable for use in the eye clinic and present data on its reliability and the distribution of stereo thresholds in adults.

**Methods:**

Test stimuli were four dynamic random-dot stereograms, one of which contained a disparate target. Feedback was given after each trial presentation. A Bayesian adaptive staircase adjusted target disparity. Threshold was estimated from the mean of the posterior distribution after 20 responses. Viewing distance was monitored via a forehead sticker viewed by the tablet's front camera, and screen parallax was adjusted dynamically so as to achieve the desired retinal disparity.

**Results:**

The tablet must be viewed at a distance of greater than ∼35 cm to produce a good depth percept. Log thresholds were roughly normally distributed with a mean of 1.75 log_10_ arcsec = 56 arcsec and SD of 0.34 log_10_ arcsec = a factor of 2.2. The standard deviation agrees with previous studies, but ASTEROID thresholds are approximately 1.5 times higher than a similar stereotest on stereoscopic 3D TV or on Randot Preschool stereotests. Pearson correlation between successive tests in same observer was 0.80. Bland-Altman 95% limits of reliability were ±0.64 log_10_ arcsec = a factor of 4.3, corresponding to an SD of 0.32 log_10_ arcsec on individual threshold estimates. This is similar to other stereotests and close to the statistical limit for 20 responses.

**Conclusions:**

ASTEROID is reliable, easy, and portable and thus well-suited for clinical stereoacuity measurements.

**Translational Relevance:**

New 3D digital technology means that research-quality psychophysical measurement of stereoacuity is now feasible in the clinic.

## Introduction

Stereopsis is the most demanding binocular visual function because it requires good vision in both eyes, good oculomotor control, and the requisite cortical mechanisms to extract depth information. Accordingly, measurement of stereoacuity is the gold standard clinically for diagnosing the presence and quality of binocular vision.^1^ Several clinical stereotests exist, including the Randot, Randot Preschool, Frisby, TNO, Titmus, and Lang stereotests.^2^,^3^ All of these share certain disadvantages: (1) consisting of cards or plates, they offer only a number of discrete levels; (2) they admit monocular cues, especially if the head is moved or tilted^4^,^5^; (3) there is a nonnegligible chance of passing a level by guessing.^3^ Given these limitations, these tests are not used to assess stereoacuity in nonclinical vision research. Instead, for many decades it has been standard to use computers to present arbitrary stimuli, run adaptive techniques such as Bayesian staircases, and/or fit psychometric functions to data.^6^–^8^,^9^ For these reasons, over the last few years several groups have proposed computerized stereotests that aim to bring laboratory-quality psychophysics to the clinic.

The Freiburg Stereoacuity Test^10^ uses 3D shutter glasses to present a stereotest consisting of a two-alternative disparity-discrimination task of a line stereogram. They used a PEST (parameter estimation by sequential testing)^11^ staircase to estimate stereo threshold from 100 presentations. Hwang and colleagues,^12^–^14^ from Seoul National University College of Medicine, also used shutter glasses to present a stereotest consisting of a four-alternative disparity-detection task in a random-dot pattern. They used a classic clinical paradigm in which at least two out of three presentations at a given level must be answered correctly in order to progress. Breyer et al.^15^ used an autostereoscopic monitor along with eye tracking to measure whether children successfully fixated a disparate random-dot target at one of four possible locations. This was intended as a test for the presence of stereovision and did not obtain a threshold measurement; the target had a constant disparity of 1200 arcsec. Hess et al.^16^ present a stereotest on an iPod using red-green anaglyph glasses, using a two-alternative disparity-discrimination task with a random-dot pattern. Threshold estimates were obtained with a staircase procedure. None of these tests are as yet commercially available, and although code for some of them is publicly available, to our knowledge none have yet been used by a laboratory other than their originators' in a scientific publication. This may be because none of the tests combine ease of use for patients (e.g., in game format, not requiring glasses) and clinicians (easy to run, no specialist knowledge of displays or set up procedure required). Thus, while there is widespread awareness of the shortcomings of current clinical stereotests, the same old-style stereotests continue to be used in the clinic.

**Figure 1:**
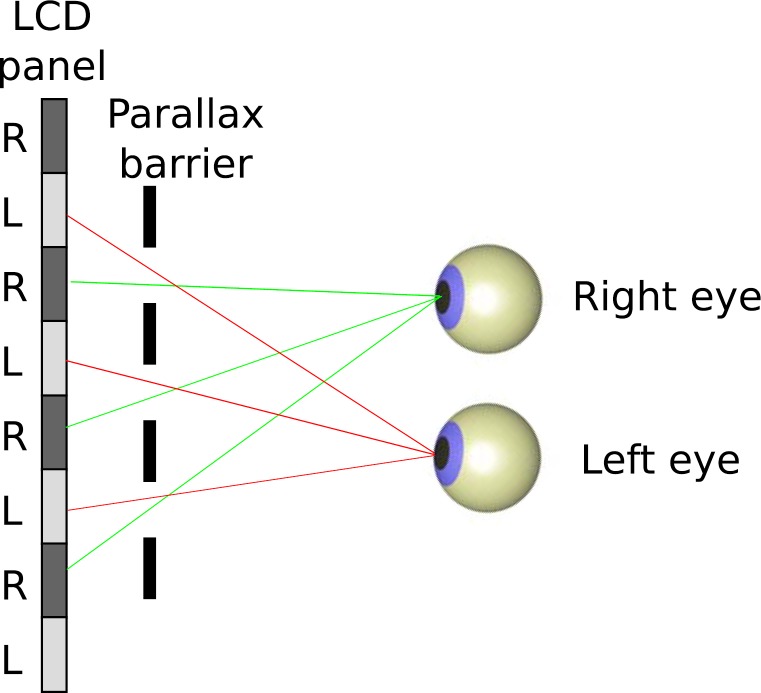
Top-down view of eyes viewing a parallax-barrier autostereoscopic display. Image reproduced from Figure 1b of Serrano-Pedraza et al.^4^

Here, we describe a new clinical stereotest that we have developed to overcome these limitations. We refer to this as ASTEROID, short for Accurate STEReotest. ASTEROID is presented on a tablet computer, so it is lightweight and portable, suitable for use in the clinic. It uses autostereo technology so that no 3D glasses are required (Fig. 1). This is a particular advantage for children, who may be unwilling to wear 3D glasses, or conversely, be more interested in playing with the glasses than doing the test. It uses a dynamic random-dot stereogram to eliminate monocular cues and present the most rigorous test of stereopsis. It uses a four-alternative forced-choice (4AFC) task, which provides more information per response than a two-alternative design, while still being simple and quick for children to perform.^17^ The computer controls the disparity via a Bayesian adaptive staircase, enabling a threshold to be obtained with high statistical efficiency. The front camera is used to monitor the viewing distance and correct the stimulus for changes in viewing distance, meaning that patients are free to hold the device how they wish rather than requiring clinician intervention to maintain the correct distance. Most importantly, the stereotest task is embedded in a fun game that uses colors, sounds, and animation to keep children engaged and responsive.^18^ We believe that this combination of features offers unique advantages, which are not achieved either by current clinical stereotests or the research tests described in the previous paragraphs.

In this paper, we give a complete description of the ASTEROID stereotest and present numerical simulations justifying our design decisions. We compare it against a laboratory psychophysics version of the test presented on a stereoscopic 3D television and against the Randot Preschool stereotest, and we present the distribution of results obtained in a nonclinical adult population. We examine its test-retest reliability in adult observers and show that it performs favorably compared to other clinical stereotests with a similar test duration.

## Methods

### ASTEROID Stereotest

#### Hardware

The ASTEROID stereotest was run on a 10.1-inch 3D tablet computer (Commander 3D, Toronto, Canada), with the specifications detailed in Table 1.

**Table 1:** Specifications of Commander 3D Autostereo Tablet Used for the ASTEROID Stereotest


Device weight	620 g
Device width	26.2 cm
Device height	17 cm
Device thickness	1 cm
Screen width	21.9 cm
Screen height	13.7 cm
Screen resolution	1920 × 1200 pixels
Physical size of 1 pixel	0.114 mm
Processor	1.5 GHz dual-core, ARM Cortex A9 TI OMAP4470
Operating system	Android 4.0.4
Front camera	2 MP

#### Disparity on a Column-Interleaved Autostereoscopic Display

This tablet has parallax-barrier autostereoscopic 3D, meaning that when 3D mode is enabled, alternate columns of pixels are visible either to the right or to the left eye (Fig. 1). Stereoscopic images can thus be displayed in column-interleaved mode, without the need for the viewer to wear special glasses. In principle, the half-images (i.e., the separate images for left and right eyes, which together define the stereoscopic image) are simply divided and interleaved column by column. However, some care needs to be taken with the definition of disparity in this case, as we will now discuss.

**Figure 2:**
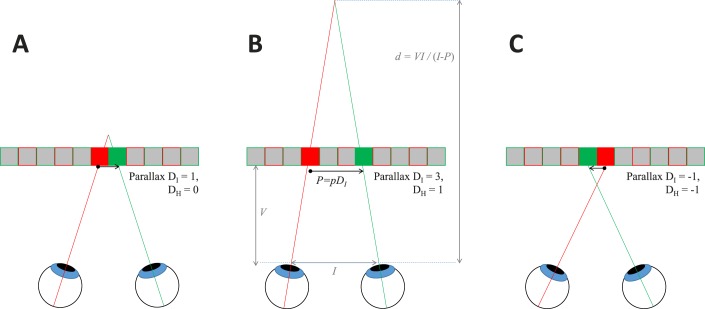
Parallax on the column-interleaved display. We use the notation of Serrano-Pedraza et al.,^4^ where D_I_ refers to the parallax in physical pixels and D_H_ to parallax in pixels of the half-images. (A) The minimum possible parallax, where corresponding points are adjacent pixels (D_I_ = 1, D_H_ = 0). (B) One H-pixel of positive parallax (D_I_ = 3, D_H_ = 1). (C) One H-pixel of negative parallax (D_I_ = −1, D_H_ = −1). P is the width of one physical pixel, so the screen parallax P is P = pD_I_. The distance of the virtual object from the viewer is d = VI/(I − P), where V is the viewing distance and I is the interocular distance. When the parallax is small compared to the interocular distance, rather than exaggerated as shown here for clarity, a parallax change of ΔP causes a change in virtual distance Δd ≈ ΔP V/I.

In other stereo displays, when the left and right half-images are identical, there is zero disparity, and the depicted image lies in the plane of the display screen. In column-interleaved displays, the left and right half-images are necessarily shifted horizontally by 1 pixel on the display; thus, even when the half-images are identical, the stereo image is slightly off the screen plane. Figure 2 shows this, using the notation developed in Serrano-Pedraza et al.^4^, where *D*_I_ refers to the parallax in physical pixels of the screen and *D*_H_ refers to parallax in pixels of the half-images we seek to depict, as discussed by Serrano-Pedraza et al.^4^ These are related by Equation 1 (also, equation 1 of Serrano-Pedraza et al.^4^)

\newcommand{\bialpha}{\boldsymbol{\alpha}}\newcommand{\bibeta}{\boldsymbol{\beta}}\newcommand{\bigamma}{\boldsymbol{\gamma}}\newcommand{\bidelta}{\boldsymbol{\delta}}\newcommand{\bivarepsilon}{\boldsymbol{\varepsilon}}\newcommand{\bizeta}{\boldsymbol{\zeta}}\newcommand{\bieta}{\boldsymbol{\eta}}\newcommand{\bitheta}{\boldsymbol{\theta}}\newcommand{\biiota}{\boldsymbol{\iota}}\newcommand{\bikappa}{\boldsymbol{\kappa}}\newcommand{\bilambda}{\boldsymbol{\lambda}}\newcommand{\bimu}{\boldsymbol{\mu}}\newcommand{\binu}{\boldsymbol{\nu}}\newcommand{\bixi}{\boldsymbol{\xi}}\newcommand{\biomicron}{\boldsymbol{\micron}}\newcommand{\bipi}{\boldsymbol{\pi}}\newcommand{\birho}{\boldsymbol{\rho}}\newcommand{\bisigma}{\boldsymbol{\sigma}}\newcommand{\bitau}{\boldsymbol{\tau}}\newcommand{\biupsilon}{\boldsymbol{\upsilon}}\newcommand{\biphi}{\boldsymbol{\phi}}\newcommand{\bichi}{\boldsymbol{\chi}}\newcommand{\bipsi}{\boldsymbol{\psi}}\newcommand{\biomega}{\boldsymbol{\omega}}\begin{equation}\tag{1}{D_{\rm{I}}} = 2{D_{\rm{H}}} + 1.\end{equation}

When the left and right half-images are identical, that is *D*_H_ = 0, the parallax is *D*_I_ = 1 physical pixel (Fig. 2A).

The parallax in centimeters is

\newcommand{\bialpha}{\boldsymbol{\alpha}}\newcommand{\bibeta}{\boldsymbol{\beta}}\newcommand{\bigamma}{\boldsymbol{\gamma}}\newcommand{\bidelta}{\boldsymbol{\delta}}\newcommand{\bivarepsilon}{\boldsymbol{\varepsilon}}\newcommand{\bizeta}{\boldsymbol{\zeta}}\newcommand{\bieta}{\boldsymbol{\eta}}\newcommand{\bitheta}{\boldsymbol{\theta}}\newcommand{\biiota}{\boldsymbol{\iota}}\newcommand{\bikappa}{\boldsymbol{\kappa}}\newcommand{\bilambda}{\boldsymbol{\lambda}}\newcommand{\bimu}{\boldsymbol{\mu}}\newcommand{\binu}{\boldsymbol{\nu}}\newcommand{\bixi}{\boldsymbol{\xi}}\newcommand{\biomicron}{\boldsymbol{\micron}}\newcommand{\bipi}{\boldsymbol{\pi}}\newcommand{\birho}{\boldsymbol{\rho}}\newcommand{\bisigma}{\boldsymbol{\sigma}}\newcommand{\bitau}{\boldsymbol{\tau}}\newcommand{\biupsilon}{\boldsymbol{\upsilon}}\newcommand{\biphi}{\boldsymbol{\phi}}\newcommand{\bichi}{\boldsymbol{\chi}}\newcommand{\bipsi}{\boldsymbol{\psi}}\newcommand{\biomega}{\boldsymbol{\omega}}\begin{equation}\tag{2}P = p{D_{\rm{I}}},\!\end{equation}

where *p* is the size of a physical pixel in centimeters (Fig. 2). The geometrical distance from the viewer of a virtual object with parallax *P* is

\newcommand{\bialpha}{\boldsymbol{\alpha}}\newcommand{\bibeta}{\boldsymbol{\beta}}\newcommand{\bigamma}{\boldsymbol{\gamma}}\newcommand{\bidelta}{\boldsymbol{\delta}}\newcommand{\bivarepsilon}{\boldsymbol{\varepsilon}}\newcommand{\bizeta}{\boldsymbol{\zeta}}\newcommand{\bieta}{\boldsymbol{\eta}}\newcommand{\bitheta}{\boldsymbol{\theta}}\newcommand{\biiota}{\boldsymbol{\iota}}\newcommand{\bikappa}{\boldsymbol{\kappa}}\newcommand{\bilambda}{\boldsymbol{\lambda}}\newcommand{\bimu}{\boldsymbol{\mu}}\newcommand{\binu}{\boldsymbol{\nu}}\newcommand{\bixi}{\boldsymbol{\xi}}\newcommand{\biomicron}{\boldsymbol{\micron}}\newcommand{\bipi}{\boldsymbol{\pi}}\newcommand{\birho}{\boldsymbol{\rho}}\newcommand{\bisigma}{\boldsymbol{\sigma}}\newcommand{\bitau}{\boldsymbol{\tau}}\newcommand{\biupsilon}{\boldsymbol{\upsilon}}\newcommand{\biphi}{\boldsymbol{\phi}}\newcommand{\bichi}{\boldsymbol{\chi}}\newcommand{\bipsi}{\boldsymbol{\psi}}\newcommand{\biomega}{\boldsymbol{\omega}}\begin{equation}\tag{3}d = VI/\left( {I - P} \right),\!\end{equation}

where *I* is the interocular distance and *V* is the viewing distance, that is, the distance from the viewer to the screen, marked in Figure 2B.

To obtain an angular disparity of *Δ* arcsec, the H parallax must be

\newcommand{\bialpha}{\boldsymbol{\alpha}}\newcommand{\bibeta}{\boldsymbol{\beta}}\newcommand{\bigamma}{\boldsymbol{\gamma}}\newcommand{\bidelta}{\boldsymbol{\delta}}\newcommand{\bivarepsilon}{\boldsymbol{\varepsilon}}\newcommand{\bizeta}{\boldsymbol{\zeta}}\newcommand{\bieta}{\boldsymbol{\eta}}\newcommand{\bitheta}{\boldsymbol{\theta}}\newcommand{\biiota}{\boldsymbol{\iota}}\newcommand{\bikappa}{\boldsymbol{\kappa}}\newcommand{\bilambda}{\boldsymbol{\lambda}}\newcommand{\bimu}{\boldsymbol{\mu}}\newcommand{\binu}{\boldsymbol{\nu}}\newcommand{\bixi}{\boldsymbol{\xi}}\newcommand{\biomicron}{\boldsymbol{\micron}}\newcommand{\bipi}{\boldsymbol{\pi}}\newcommand{\birho}{\boldsymbol{\rho}}\newcommand{\bisigma}{\boldsymbol{\sigma}}\newcommand{\bitau}{\boldsymbol{\tau}}\newcommand{\biupsilon}{\boldsymbol{\upsilon}}\newcommand{\biphi}{\boldsymbol{\phi}}\newcommand{\bichi}{\boldsymbol{\chi}}\newcommand{\bipsi}{\boldsymbol{\psi}}\newcommand{\biomega}{\boldsymbol{\omega}}\begin{equation}\tag{4}{D_{\rm H}} = {V \over {2p}}\tan \left( {{{\pi {\it{\Delta}} } \over {180 \times 3600}}} \right),\!\end{equation}

where *D*_H_ is screen parallax in H-pixels, 2*p* is the width of one H-pixel (i.e., twice the width *p* of one physical I-pixel on the screen), and *V* is the viewing distance in the same units as *p*. The term 1/3600 converts from arcseconds to degrees and π/180 converts from degrees to radians.

The minimum whole-pixel parallax change is 1 H-pixel, which implies a change of 2 I-pixels (compare Fig. 2A versus Figs. 2B and 2C, and note that when *D*_H_ changes by 1, *D*_I_ changes by 2). This corresponds to an angular disparity of 2*p/V* radians. This is the minimum relative disparity between the target and the background that can be depicted without special techniques to achieve subpixel disparity (compare Fig. 2A versus Fig. 2B, where disparity differs by a single H-pixel). On the Commander 3D, each pixel has a physical width of *p* = 0.114 mm and therefore subtends an angle of 0.0164° = 59 arcsec at a viewing distance of 40 cm. The minimum whole-pixel relative disparity is therefore 118 arcsec at 40 cm. Note that this is twice what one might imagine based on the screen resolution without the additional complication of column interleaving.

#### Stimulus and Task

**Figure 3:**
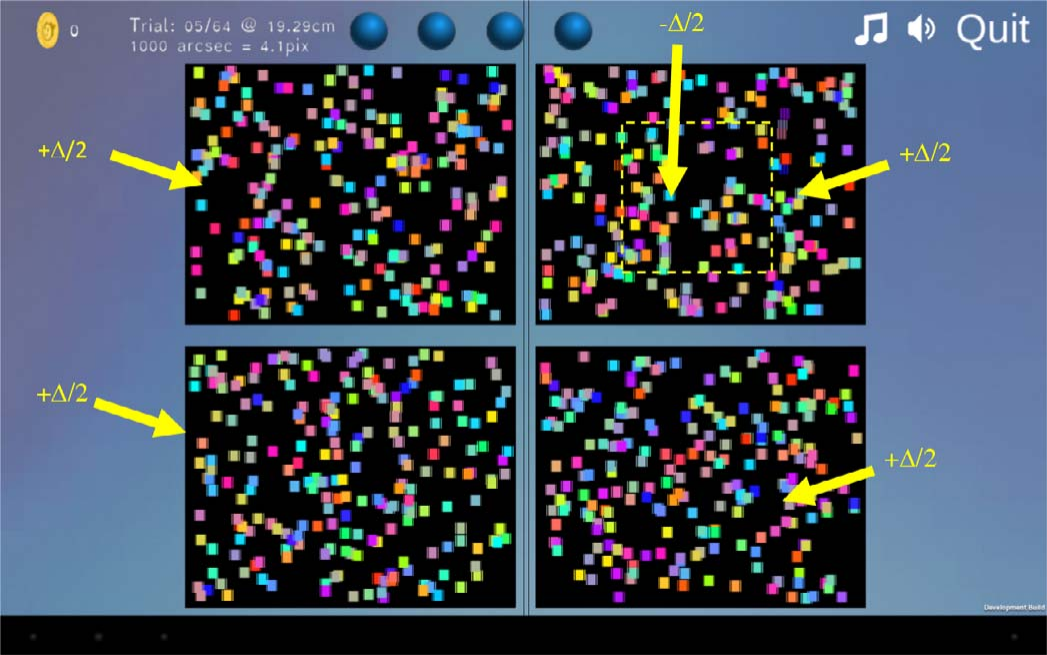
Screenshot showing the test stimulus, with four patches of random-dot patterns. Three of these have a uniform disparity of +Δ/2, depicting a planar surface behind the screen, whereas the top-right patch contains a “target” region with disparity −Δ/2 (in front of the screen) on a background surface with disparity −Δ/2. The relative disparity between target and background is thus Δ. The yellow symbols, including the dashed square indicating the target, are shown for illustration and were not present in the stimulus.

As shown in Figure 3, the test stimulus consists of four patches of dynamic random-dot patterns, each 615 × 490 physical pixels on the screen. The individual dots are 20 pixels vertically and 9 pixels horizontally in each half-image, thus after interleaving they appear square. Three of the patches depict flat surfaces while the fourth has a square “target” (285 × 285 I-pixels) floating in front of a background. The task is to touch the patch containing the target. Thus, the task requires the user only to detect disparity, not to discriminate its sign. The four-alternative design was chosen because our previous work has demonstrated that this is most efficient for measuring stereo thresholds in children.^19^ The dots were made colored rather than white simply to make the stimulus more attractive. Since the luminance pattern alone unambiguously defined disparity, there is no reason to expect the color to affect stereo thresholds.^20^

Task difficulty is controlled by the relative disparity *Δ* between the target and the background. In order to avoid monocular cues to the location of the target, this was always applied symmetrically; that is, the background had disparity *Δ*/2 and the target had disparity −*Δ*/2, as indicated in Figure 3. Serrano-Pedraza et al.^4^ provide an in-depth discussion of this and other techniques we used to eliminate monocular artifacts in this stimulus.

The stimulus was dynamically updated with new patterns of random dots. The update was as rapid as the tablet could manage, generally around 10 frames per second. This was fast enough to avoid monocular artifacts created by rapid movements of the head relative to the tablet, since each dot vanished before its monocular motion could be detected.^4^ The stimulus continued until a response was made.

#### Applying Disparity

Given the desired relative disparity *Δ* between the target and background, we first compute the desired screen parallax in pixels of each eye's half-image, H-pixels *D*_H_, according to Equation 4. We scatter dots uniformly across each patch. To apply the background parallax of +*D*_H_/2, we add *D*_H_/4 to the *x*-coordinate of each dot in the right eye and subtract *D*_H_/4 from the *x*-coordinate of each dot in the left eye. In the same way, we generated a patch of dots for the target, but this time we subtract *D*_H_/4 from the *x*-coordinate of each dot in the right eye and added (*D*_H_/4 − 1) to the *x*-coordinate of each target dot in the left eye. The reason for the additional 1-pixel leftward shift in the left eye is to avoid monocular artifacts due to the column interleaving, as explained by Serrano-Pedraza.^4^ Before we draw the dots, we remove any background dots that would be occluded by the target, taking into account the shift of the target in each eye.

**Figure 4:**
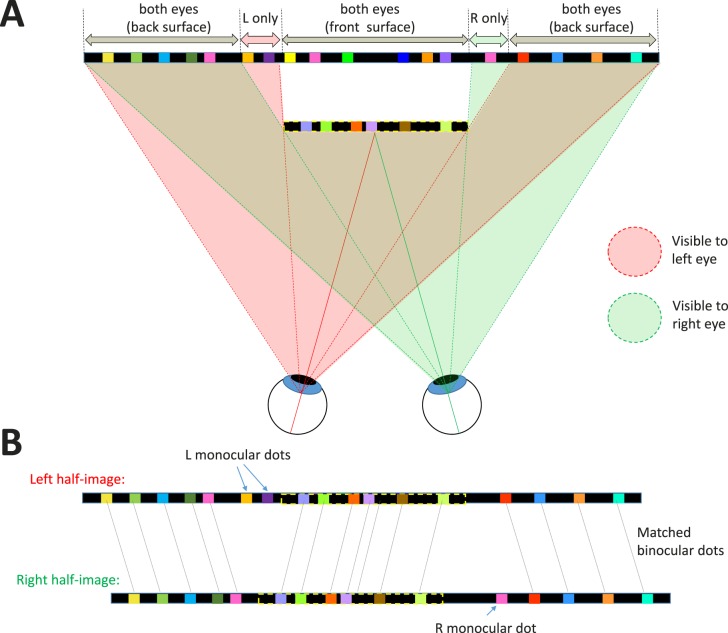
Binocular occlusion geometry for our stimulus where a target surface (outlined in dashed yellow lines) appears in front of a background. (A) The front surface occludes different regions of the background in the two eyes. The pink (green) shaded regions indicate which parts of the stimulus are visible to the left (right) eye, respectively. To the left of the front surface, there is a narrow strip of the back surface that is visible only to the left eye. Dots in this region are accordingly left-monocular, visible only to the left eye. The same applies for the right eye, for a strip to the right of the front surface. (B) Shows the resulting left- and right-eye half-images. Most dots are binocular, that is, visible in both eyes, so a disparity can be defined. Dots on the back surface have uncrossed disparity (position in the left half-image is to the left of position in the right half-image), whereas dots on the front surface have crossed disparity (position in the left half-image is to the right of position in the right half-image). The monocular dots have no matching dots in the other eye and are said to be uncorrelated. Nothing identifies the uncorrelated dots in either half-image individually; they can be detected only when the two eyes' half-images are compared.

According to the usual occlusion geometry of binocular vision, shown in Figure 4, there is a monocular strip down each side of the target. Dots falling in this strip are visible to one eye only, so the dots have no matches in the other half-image. For example in Figure 4B, the orange and purple dots labeled “L monocular dots” appear only in the left half-image; they have been removed from the right half-image to simulate their being occluded by the front surface. The width of this monocular strip is equal to the parallax. Note that in reality, with randomly placed dots, some dots might be only partly occluded; part of the dot could still be visible peeping out from behind the front surface. If we allowed this to happen in our images, the presence of such dot fragments would provide a monocular cue as to which patch contains the target. To avoid this, we remove the *whole* dot from a half-image whenever its center falls within the occluded region. Geometrically, this means that each individual dot is either entirely on the front surface or the back surface, and thus the edges of our target surface are slightly ragged, varying by up to one dot width.

In practice, the parallax is usually smaller than the width of each dot, so on most frames there will be no monocular dots. As the parallax approaches zero, monocular dots become rarer and rarer. However, when they do occur, the resulting monocular dots are quite salient to the experienced observer. We found during pilot studies that experienced observers alert to this trick could detect the target no matter how small the disparity, simply by viewing the stimulus long enough to detect the occasional monocular dot. Of course, they were still using their binocular vision to do this, but the task is then no longer measuring stereoacuity. To remove the possibility of this strategy, monocular dots were removed when the disparity was <500 arcsec. That is, no dots were drawn in the occluded regions. In principle, this generates a monocular density artifact, but since the occluded regions were so narrow, we found that this cue was undetectable at disparities <500 arcsec, even for our most experienced and wily observers.

#### Obtaining Subpixel Disparities

As noted above, the minimum relative disparity depictable in whole pixels on the Commander 3D tablet is 100 arcsec at a viewing distance of 47 cm. To approximate subpixel disparities, we explored spatiotemporal dithering and antialiasing. In dithering, to simulate a parallax of 0.6 pixels, we gave 60% of the dots a parallax of 1 pixel and 40% a parallax of 0 pixels. In our dynamically updated random-dot patterns, this approximated a difficulty level intermediate between 1 and 0 pixels. Dithering was used in ASTEROID versions 0.934 to 0.948. In antialiasing, to render a dot edge at 5.6 pixels, we give pixel number 5 the full color of the dot, but pixel number 6 is colored with a mixture of 60% of the dot's color and 40% of the black background.^4^ Antialiasing was used in ASTEROID versions up to 0.933 and again from version 1.00. Both approaches have their pros and cons. Antialiasing is more accurate when the point-spread function of the eye is large enough relative to a pixel, since it then becomes indistinguishable from the desired stimulus.^21^ However, this requires accurate luminance linearization. This would be hard to achieve on individual tablets, especially given that users might alter the display brightness and contrast, and we did not attempt it. Dithering is less accurate but potentially more robust. In practice, we did not find a significant difference between these two approaches, given the other sources of error discussed in the results. We have therefore combined results from all versions in this paper.

#### Monitoring Viewing Distance

**Figure 5:**
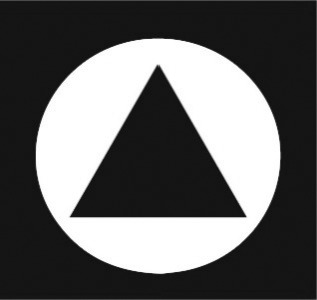
The sticker used as a target for distance tracking. Its dimensions are 37 mm wide by 35 mm high.

Viewing distance was monitored automatically using the device's front camera. The participants wore a sticker (Fig. 5) on their foreheads, bearing a distinctive high-contrast design chosen to be easily detectable via standard computer vision algorithms. The program is calibrated so that it can estimate viewing distance based on the apparent size of the sticker in the camera image. Each time the dynamic random-dot pattern was updated, the program used the current estimate of viewing distance in order to calculate the screen parallax (in pixels) required to produce the desired retinal disparity (in seconds of arc) according to Equation 4. The aim was that if the participant moved the tablet farther/closer to him or her, the parallax would be increased/decreased in order to keep the retinal disparity the same. This process was not 100% reliable; the sticker was occasionally not identified due to issues such as reflections, hair, how the person was holding the tablet, and so forth. In such cases, the last valid viewing distance estimate was used. Where no estimate could be obtained at all, a default of 25 cm was used. Other than in experiment 1, only tests in which the mean reported distance estimate was over 35 cm were included in the analysis.

#### Accelerometry

The tablet's accelerometer was monitored throughout the test. If the tablet was shaken during a stimulus presentation or rotated so that it was in portrait mode (where the parallax-barrier autostereo no longer works), the dynamic random dots vanished so that the stimulus screen showed four blank black patches. As soon as the movement stopped or the landscape view was restored, the dots returned. This discouraged participants from holding the tablet inappropriately. Note that in other stereotests that use linearly polarizing filters, for example Randot and Randot Preschool stereotests, a 45° rotation produces 100% cross talk (and consequently artifacts that can be used to perform the test monocularly), while a 90° rotation converts horizontal disparity into vertical disparity. In ASTEROID, rotating the device prevents the parallax barrier from working as intended. Rotations can produce cross talk (but no artifacts that could be used to perform the test monocularly) or mean that both eyes see the same image (a flat 2D display, so again no monocular artifacts). Thus, rotating the device can only impair performance. Blanking the display was not done to avoid artifacts, but simply to signal participants that they are holding the device incorrectly.

#### The Task

In accordance with common usage in psychophysical literature, we refer to each presentation of the stimulus, requiring a single response from the participant, as *a trial*. We refer to a sequence of several trials, resulting in a threshold estimate, as *a test*.

##### Practice Trials and Nonstereo Cue

The initial four trials contained a nonstereo cue to the location of the target: the target was outlined with white lines. After each correct answer, the alpha-value^25^ of the target was reduced, making it more transparent, until after four correct answers it vanished and the target could be detected only via its stereoscopic disparity. Usually, all subsequent trials would be stereo only. However, as described below, the nonstereo cue could reappear in subsequent trials if the threshold estimate suggested that the participant was stereoblind. The aim was to keep performance at 75% correct, regardless of the participant's stereoacuity.

##### Feedback

After the participant gives a response by touching one of the patches, the dynamic random dots disappear. Feedback was provided via an animation. If the participant touched the patch containing the target, a box appears at the target location that then opens to reveal an object, while colored “confetti” rains down from the center of the screen and applause sounds. If the participant touched an incorrect patch, a box appears in the correct target location, but does not open, while the device buzzes briefly.

##### Random Order

**Figure 6:**
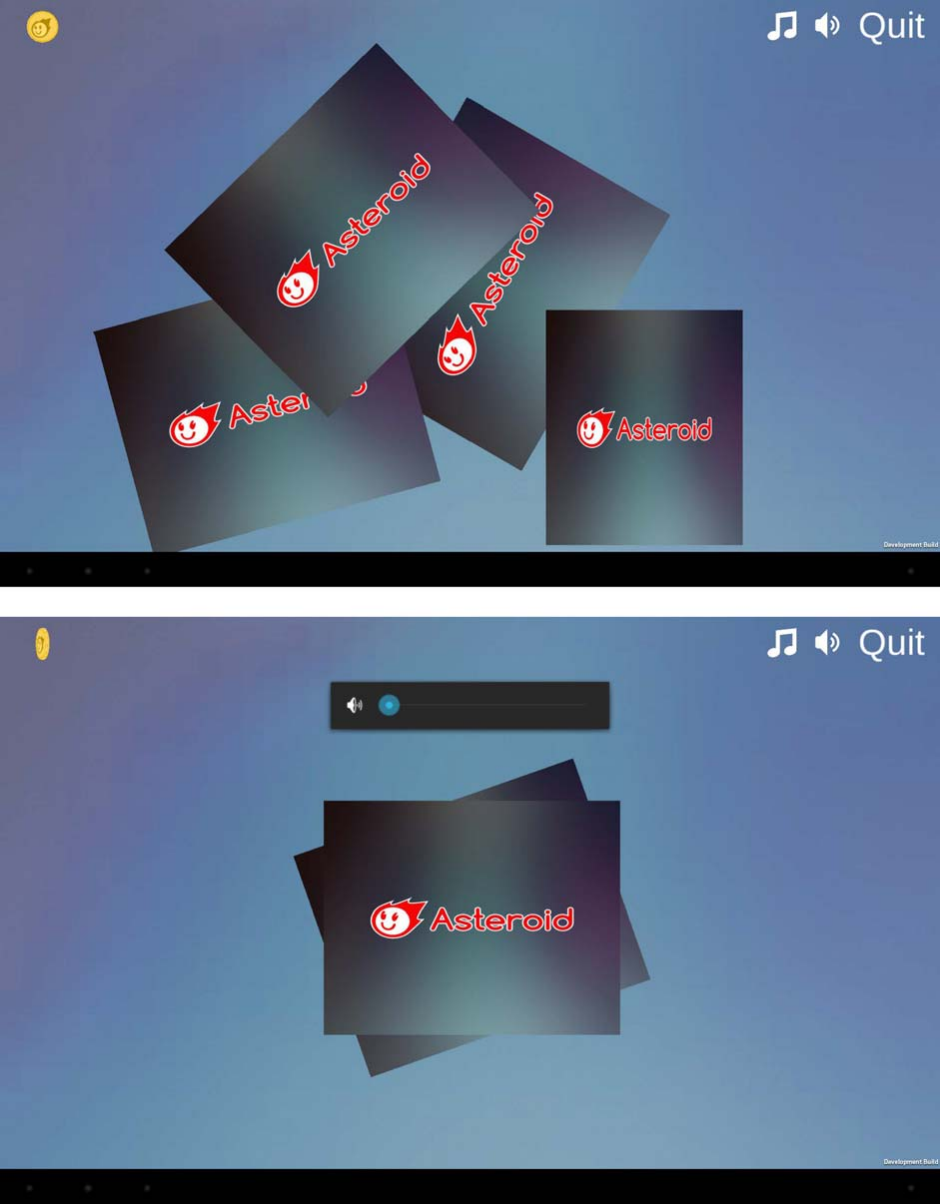
Screenshots from the animation showing the four stimuli being shuffled and dealt out again as a cue to the random stimulus location.

The target could be in any of the four possible locations, chosen at random with equal probability and no memory. That is, the target's location on one trial did not make it any more or less likely to appear in that location on the next trial. As a visual cue to convey this to participants, after the feedback animation following each trial, the four patches turned over like playing cards to reveal the ASTEROID logo, were gathered into the center of the screen, and dealt out again face down to the four locations. They then turned over to reveal the dynamic random-dot patterns (Figure 6).

#### Game Themes

ASTEROID can run either in standard mode, in which the trials are simply presented in sequence, or as a game, in which the trials are interspersed with brief animated scenes describing a story and the task is represented as helping game characters find something they need. There are four game themes to choose from, designed to appeal to children of different ages and genders: Shape World, Chicken Farm, Fairy Forest, and Soccer Match. The games were developed by Fluid Pixel Ltd (Newcastle, UK).

#### The Psychometric Function

Using a standard signal detection approach, we model performance of our disparity-detection task as follows. We envisage the disparity target as generating a signal inside the observer's visual system, representing the logarithm of the relative disparity. This signal is also subject to noise, which we model as drawn from a probability density function:

\newcommand{\bialpha}{\boldsymbol{\alpha}}\newcommand{\bibeta}{\boldsymbol{\beta}}\newcommand{\bigamma}{\boldsymbol{\gamma}}\newcommand{\bidelta}{\boldsymbol{\delta}}\newcommand{\bivarepsilon}{\boldsymbol{\varepsilon}}\newcommand{\bizeta}{\boldsymbol{\zeta}}\newcommand{\bieta}{\boldsymbol{\eta}}\newcommand{\bitheta}{\boldsymbol{\theta}}\newcommand{\biiota}{\boldsymbol{\iota}}\newcommand{\bikappa}{\boldsymbol{\kappa}}\newcommand{\bilambda}{\boldsymbol{\lambda}}\newcommand{\bimu}{\boldsymbol{\mu}}\newcommand{\binu}{\boldsymbol{\nu}}\newcommand{\bixi}{\boldsymbol{\xi}}\newcommand{\biomicron}{\boldsymbol{\micron}}\newcommand{\bipi}{\boldsymbol{\pi}}\newcommand{\birho}{\boldsymbol{\rho}}\newcommand{\bisigma}{\boldsymbol{\sigma}}\newcommand{\bitau}{\boldsymbol{\tau}}\newcommand{\biupsilon}{\boldsymbol{\upsilon}}\newcommand{\biphi}{\boldsymbol{\phi}}\newcommand{\bichi}{\boldsymbol{\chi}}\newcommand{\bipsi}{\boldsymbol{\psi}}\newcommand{\biomega}{\boldsymbol{\omega}}\begin{equation}\tag{5}\phi \left( \varepsilon \right) = {{\exp \left( \varepsilon \right)} \over {{{\left( {1 + \exp \left( \varepsilon \right)} \right)}^2}}}.\end{equation}

This is the first derivative of the logistic function, which closely resembles a Gaussian function. The noisy signal available to the visual system on each trial is therefore

$$\newcommand{\bialpha}{\boldsymbol{\alpha}}\newcommand{\bibeta}{\boldsymbol{\beta}}\newcommand{\bigamma}{\boldsymbol{\gamma}}\newcommand{\bidelta}{\boldsymbol{\delta}}\newcommand{\bivarepsilon}{\boldsymbol{\varepsilon}}\newcommand{\bizeta}{\boldsymbol{\zeta}}\newcommand{\bieta}{\boldsymbol{\eta}}\newcommand{\bitheta}{\boldsymbol{\theta}}\newcommand{\biiota}{\boldsymbol{\iota}}\newcommand{\bikappa}{\boldsymbol{\kappa}}\newcommand{\bilambda}{\boldsymbol{\lambda}}\newcommand{\bimu}{\boldsymbol{\mu}}\newcommand{\binu}{\boldsymbol{\nu}}\newcommand{\bixi}{\boldsymbol{\xi}}\newcommand{\biomicron}{\boldsymbol{\micron}}\newcommand{\bipi}{\boldsymbol{\pi}}\newcommand{\birho}{\boldsymbol{\rho}}\newcommand{\bisigma}{\boldsymbol{\sigma}}\newcommand{\bitau}{\boldsymbol{\tau}}\newcommand{\biupsilon}{\boldsymbol{\upsilon}}\newcommand{\biphi}{\boldsymbol{\phi}}\newcommand{\bichi}{\boldsymbol{\chi}}\newcommand{\bipsi}{\boldsymbol{\psi}}\newcommand{\biomega}{\boldsymbol{\omega}}x = {\log _{10}}{{{\it{\Delta}} }} + {\varepsilon \over b}{\rm {,}}$$

where *ε* is the noise on that trial, a random variable drawn from *P*_noise_, and *b* is the inverse of the noise amplitude. Higher values of *b* indicate lower noise and therefore greater sensitivity. Our model assumes that the target is detected if and only if this noisy signal exceeds an internal detection threshold *A*,^22^,^23^ that is, the noise *ε* exceeds *b*(*A* − log_10_*Δ*). The probability that this occurs is

$$\newcommand{\bialpha}{\boldsymbol{\alpha}}\newcommand{\bibeta}{\boldsymbol{\beta}}\newcommand{\bigamma}{\boldsymbol{\gamma}}\newcommand{\bidelta}{\boldsymbol{\delta}}\newcommand{\bivarepsilon}{\boldsymbol{\varepsilon}}\newcommand{\bizeta}{\boldsymbol{\zeta}}\newcommand{\bieta}{\boldsymbol{\eta}}\newcommand{\bitheta}{\boldsymbol{\theta}}\newcommand{\biiota}{\boldsymbol{\iota}}\newcommand{\bikappa}{\boldsymbol{\kappa}}\newcommand{\bilambda}{\boldsymbol{\lambda}}\newcommand{\bimu}{\boldsymbol{\mu}}\newcommand{\binu}{\boldsymbol{\nu}}\newcommand{\bixi}{\boldsymbol{\xi}}\newcommand{\biomicron}{\boldsymbol{\micron}}\newcommand{\bipi}{\boldsymbol{\pi}}\newcommand{\birho}{\boldsymbol{\rho}}\newcommand{\bisigma}{\boldsymbol{\sigma}}\newcommand{\bitau}{\boldsymbol{\tau}}\newcommand{\biupsilon}{\boldsymbol{\upsilon}}\newcommand{\biphi}{\boldsymbol{\phi}}\newcommand{\bichi}{\boldsymbol{\chi}}\newcommand{\bipsi}{\boldsymbol{\psi}}\newcommand{\biomega}{\boldsymbol{\omega}}{P_{\rm det}}\left( {\rm{\Delta }} \right) = \int _{b\left( {A - {\rm{lo}}{{\rm{g}}_{10}}{\rm{{\it{\Delta}} }}} \right)}^\infty \phi \left( \varepsilon \right)d\epsilon {\rm {.}}$$

This integrates to

\newcommand{\bialpha}{\boldsymbol{\alpha}}\newcommand{\bibeta}{\boldsymbol{\beta}}\newcommand{\bigamma}{\boldsymbol{\gamma}}\newcommand{\bidelta}{\boldsymbol{\delta}}\newcommand{\bivarepsilon}{\boldsymbol{\varepsilon}}\newcommand{\bizeta}{\boldsymbol{\zeta}}\newcommand{\bieta}{\boldsymbol{\eta}}\newcommand{\bitheta}{\boldsymbol{\theta}}\newcommand{\biiota}{\boldsymbol{\iota}}\newcommand{\bikappa}{\boldsymbol{\kappa}}\newcommand{\bilambda}{\boldsymbol{\lambda}}\newcommand{\bimu}{\boldsymbol{\mu}}\newcommand{\binu}{\boldsymbol{\nu}}\newcommand{\bixi}{\boldsymbol{\xi}}\newcommand{\biomicron}{\boldsymbol{\micron}}\newcommand{\bipi}{\boldsymbol{\pi}}\newcommand{\birho}{\boldsymbol{\rho}}\newcommand{\bisigma}{\boldsymbol{\sigma}}\newcommand{\bitau}{\boldsymbol{\tau}}\newcommand{\biupsilon}{\boldsymbol{\upsilon}}\newcommand{\biphi}{\boldsymbol{\phi}}\newcommand{\bichi}{\boldsymbol{\chi}}\newcommand{\bipsi}{\boldsymbol{\psi}}\newcommand{\biomega}{\boldsymbol{\omega}}\begin{equation}\tag{6}{P_{\rm det}}\left( {\rm{\Delta }} \right) = {1 \over {\left( {1 + \exp \left[ {b\left( {{{A}} - {{\log }_{10}}{\rm{{\it{\Delta}} }}} \right)} \right]} \right)}}.\end{equation}

Thus, in this model, the probability that the observer detects the target is a logistic function of disparity. Of course, we do not ask participants directly whether they detect the target, since this would introduce additional issues about the criterion they adopt, how conservative they are, and so forth. Instead, we ask them to choose which patch contains the target. An ideal observer would answer correctly either when they perceive the target *or* when they cannot perceive the target but guess correctly. Thus, for an ideal observer, the probability of a correct answer would be Ψ = *P*_det_ + (1 − *P*_det_)*g*, where *g* is the probability of answering correctly by guessing (*g* = 0.25 on our four-alternative task). However, humans are not ideal, and therefore even if the signal is well above threshold, there is a finite probability *λ* that the observer will give the wrong answer anyway, for example, because their attention wandered or their finger slipped. Thus, we follow established practice and alter the previous expression to Ψ = (1 − *λ*)*P*_det_ + (1 − *P*_det_)*g* = *g* + (1 − *λ* − *g*) *P*_det_.

Substituting for the probability of perceiving the target from Equation 6, we obtain the following expression for the probability of answering correctly:

\newcommand{\bialpha}{\boldsymbol{\alpha}}\newcommand{\bibeta}{\boldsymbol{\beta}}\newcommand{\bigamma}{\boldsymbol{\gamma}}\newcommand{\bidelta}{\boldsymbol{\delta}}\newcommand{\bivarepsilon}{\boldsymbol{\varepsilon}}\newcommand{\bizeta}{\boldsymbol{\zeta}}\newcommand{\bieta}{\boldsymbol{\eta}}\newcommand{\bitheta}{\boldsymbol{\theta}}\newcommand{\biiota}{\boldsymbol{\iota}}\newcommand{\bikappa}{\boldsymbol{\kappa}}\newcommand{\bilambda}{\boldsymbol{\lambda}}\newcommand{\bimu}{\boldsymbol{\mu}}\newcommand{\binu}{\boldsymbol{\nu}}\newcommand{\bixi}{\boldsymbol{\xi}}\newcommand{\biomicron}{\boldsymbol{\micron}}\newcommand{\bipi}{\boldsymbol{\pi}}\newcommand{\birho}{\boldsymbol{\rho}}\newcommand{\bisigma}{\boldsymbol{\sigma}}\newcommand{\bitau}{\boldsymbol{\tau}}\newcommand{\biupsilon}{\boldsymbol{\upsilon}}\newcommand{\biphi}{\boldsymbol{\phi}}\newcommand{\bichi}{\boldsymbol{\chi}}\newcommand{\bipsi}{\boldsymbol{\psi}}\newcommand{\biomega}{\boldsymbol{\omega}}\begin{equation}\tag{7}{\rm{\Psi }}\left( {\rm{{\it{\Delta}} }} \right) = g + {{\left( {1 - \lambda - g} \right)} \over {\left( {1 + \exp \left[ {b\left( {{{A}} - {{\log }_{10}}{{{\it{\Delta}} }}} \right)} \right]} \right)}}.\end{equation}

We define the task-specific stereo threshold, *θ*, to be the value of disparity *Δ* needed to obtain a particular level of performance, Θ, on our task:

\newcommand{\bialpha}{\boldsymbol{\alpha}}\newcommand{\bibeta}{\boldsymbol{\beta}}\newcommand{\bigamma}{\boldsymbol{\gamma}}\newcommand{\bidelta}{\boldsymbol{\delta}}\newcommand{\bivarepsilon}{\boldsymbol{\varepsilon}}\newcommand{\bizeta}{\boldsymbol{\zeta}}\newcommand{\bieta}{\boldsymbol{\eta}}\newcommand{\bitheta}{\boldsymbol{\theta}}\newcommand{\biiota}{\boldsymbol{\iota}}\newcommand{\bikappa}{\boldsymbol{\kappa}}\newcommand{\bilambda}{\boldsymbol{\lambda}}\newcommand{\bimu}{\boldsymbol{\mu}}\newcommand{\binu}{\boldsymbol{\nu}}\newcommand{\bixi}{\boldsymbol{\xi}}\newcommand{\biomicron}{\boldsymbol{\micron}}\newcommand{\bipi}{\boldsymbol{\pi}}\newcommand{\birho}{\boldsymbol{\rho}}\newcommand{\bisigma}{\boldsymbol{\sigma}}\newcommand{\bitau}{\boldsymbol{\tau}}\newcommand{\biupsilon}{\boldsymbol{\upsilon}}\newcommand{\biphi}{\boldsymbol{\phi}}\newcommand{\bichi}{\boldsymbol{\chi}}\newcommand{\bipsi}{\boldsymbol{\psi}}\newcommand{\biomega}{\boldsymbol{\omega}}\begin{equation}\tag{8}\Theta = g + {{\left( {1 - \lambda - g} \right)} \over {\left( {1 + \exp \left[ {b\left( {{{A}} - {{\log }_{10}}\theta } \right)} \right]} \right)}}.\end{equation}

The stereo threshold returned by ASTEROID after the completion of each test is the estimate of *θ*.

We can rewrite Equation 7 in terms of the stereo threshold *θ* as

\newcommand{\bialpha}{\boldsymbol{\alpha}}\newcommand{\bibeta}{\boldsymbol{\beta}}\newcommand{\bigamma}{\boldsymbol{\gamma}}\newcommand{\bidelta}{\boldsymbol{\delta}}\newcommand{\bivarepsilon}{\boldsymbol{\varepsilon}}\newcommand{\bizeta}{\boldsymbol{\zeta}}\newcommand{\bieta}{\boldsymbol{\eta}}\newcommand{\bitheta}{\boldsymbol{\theta}}\newcommand{\biiota}{\boldsymbol{\iota}}\newcommand{\bikappa}{\boldsymbol{\kappa}}\newcommand{\bilambda}{\boldsymbol{\lambda}}\newcommand{\bimu}{\boldsymbol{\mu}}\newcommand{\binu}{\boldsymbol{\nu}}\newcommand{\bixi}{\boldsymbol{\xi}}\newcommand{\biomicron}{\boldsymbol{\micron}}\newcommand{\bipi}{\boldsymbol{\pi}}\newcommand{\birho}{\boldsymbol{\rho}}\newcommand{\bisigma}{\boldsymbol{\sigma}}\newcommand{\bitau}{\boldsymbol{\tau}}\newcommand{\biupsilon}{\boldsymbol{\upsilon}}\newcommand{\biphi}{\boldsymbol{\phi}}\newcommand{\bichi}{\boldsymbol{\chi}}\newcommand{\bipsi}{\boldsymbol{\psi}}\newcommand{\biomega}{\boldsymbol{\omega}}\begin{equation}\tag{9}{\rm{\Psi }}\left( {{\rm{{\it{\Delta}} }};{\rm{\theta }}} \right) = g + {{\left( {1 - \lambda - g} \right)} \over {\left( {1 + {{\left( {1\ -\ \lambda\ -\ \Theta} \right)} \over {\left( {\Theta\ -\ g} \right)}}\exp \left[ {{{b\log }_{10}}\left( {{{\theta} \over {\rm{{\it{\Delta}} }}}} \right)} \right]} \right)}}.\end{equation}

##### Selecting Parameters for the Psychometric Function

Equation 9 is our expression for the psychometric function, that is, the probability of correctly selecting the target as a function of target disparity. To summarize, *Δ* is the disparity of the target relative to the background and *θ* is the participant's task-specific stereo threshold, in arcseconds, defined as the value of *Δ* for which the probability of selecting the target is Θ (so by definition Ψ(*θ*) = Θ). In ASTEROID, Θ is chosen to be 0.75, that is, 75% correct. We discuss and justify this choice in the Results: Simulation 1. It is important to bear this in mind when comparing ASTEROID thresholds with those obtained on other tests.

The probability of selecting the target by chance, *g* (for guessing), is 0.25 in our four-alternative task. The probability of giving the wrong answer even when the stimulus is well above threshold is *λ*, conventionally called the *lapse rate*.^24^ Performance thus asymptotes at (1 − *λ*). Realistically, lapse rates might vary from as low as 0.001 to as high as 0.1 for different participants, but the precise value of the lapse rate within this range does not usually affect threshold estimates.^24^ We used *λ* = 0.03, chosen to be in the middle of the realistic range.

The parameter *b* controls how rapidly performance improves as disparity is increased. We could attempt to fit *b* for individual subjects, but this would require more trials than are clinically feasible. Thus, we fixed *b* at a value we know to be reasonable from a previous study^25^: *b* = 4.885/log_10_ arcsec.

#### Choosing Disparity Values on Each Trial

##### Introductory Phase

As described above, the test begins with practice trials containing a nonstereo (luminance) cue to the location of the target. During this introductory phase, the target and its framing cue have a disparity of *Δ* = 1000 arcsec relative to the background. This disparity is highly visible to most people with normal stereo vision.

In this introductory phase, after each correct answer, the opacity (alpha channel value^26^) of the cue frame is reduced by 0.14285, making it gradually more transparent. After each incorrect answer, the alpha value is increased by the same amount (capped at 1). After four trials have been answered correctly, the test moves out of the introduction and into the stereotest proper. If after 10 trials fewer than four trials have been answered correctly, we conclude that the participant is not capable of doing the task (whether because of poor understanding, motivation, or low vision), and the test terminates.

##### Bayesian Threshold Estimate

After leaving the introductory phase, the stereotest proper then began. We use a Bayesian framework where the participant's responses are used successively to update our estimated probability density function for their stereo log threshold, *Q_n_*(*q*). *Q_n_*(*q*)d*q* represents our estimate, after *n* trials, of the probability that the participant's log threshold is *q* (or more properly, that the log threshold lies between *q* and *q* + d*q* log_10_ arcsec). We update this after each trial based on whether the participant's response on that trial was correct or incorrect.

Bayes' theorem tells us that

\newcommand{\bialpha}{\boldsymbol{\alpha}}\newcommand{\bibeta}{\boldsymbol{\beta}}\newcommand{\bigamma}{\boldsymbol{\gamma}}\newcommand{\bidelta}{\boldsymbol{\delta}}\newcommand{\bivarepsilon}{\boldsymbol{\varepsilon}}\newcommand{\bizeta}{\boldsymbol{\zeta}}\newcommand{\bieta}{\boldsymbol{\eta}}\newcommand{\bitheta}{\boldsymbol{\theta}}\newcommand{\biiota}{\boldsymbol{\iota}}\newcommand{\bikappa}{\boldsymbol{\kappa}}\newcommand{\bilambda}{\boldsymbol{\lambda}}\newcommand{\bimu}{\boldsymbol{\mu}}\newcommand{\binu}{\boldsymbol{\nu}}\newcommand{\bixi}{\boldsymbol{\xi}}\newcommand{\biomicron}{\boldsymbol{\micron}}\newcommand{\bipi}{\boldsymbol{\pi}}\newcommand{\birho}{\boldsymbol{\rho}}\newcommand{\bisigma}{\boldsymbol{\sigma}}\newcommand{\bitau}{\boldsymbol{\tau}}\newcommand{\biupsilon}{\boldsymbol{\upsilon}}\newcommand{\biphi}{\boldsymbol{\phi}}\newcommand{\bichi}{\boldsymbol{\chi}}\newcommand{\bipsi}{\boldsymbol{\psi}}\newcommand{\biomega}{\boldsymbol{\omega}}\begin{equation}\tag{10}\Pr \left\{ {{\rm{response}}|q} \right\}\Pr \left\{ q \right\} = \Pr \left\{ {q|{\rm{response}}} \right\}\Pr \left\{ {{\rm{response}}} \right\},\end{equation}

where Pr{response|*q*} represents the probability of the observed response (correct or incorrect) on the *n*th trial, given that the threshold is *q*, Pr{*q*} the probability that the log threshold is *q*, Pr{*q*|response} the probability that the log threshold is *q* given the observed response, and Pr{response} the probability of the observed response.

The psychometric function, Ψ(*Δ*;*θ*), tells us the probability of a correct response, while (1 − Ψ(*Δ*;*θ*)) is the probability of an incorrect response, both for a threshold of *θ* = 10*^q^*. At the start of the *n*th trial, our current estimate of Pr{*q*} is *Q_n−1_*(*q*). After the *n*th trial, it has been updated to *Q_n_*(*q*) = Pr{*q*|response}. Thus, Equation 10 tells us that

\newcommand{\bialpha}{\boldsymbol{\alpha}}\newcommand{\bibeta}{\boldsymbol{\beta}}\newcommand{\bigamma}{\boldsymbol{\gamma}}\newcommand{\bidelta}{\boldsymbol{\delta}}\newcommand{\bivarepsilon}{\boldsymbol{\varepsilon}}\newcommand{\bizeta}{\boldsymbol{\zeta}}\newcommand{\bieta}{\boldsymbol{\eta}}\newcommand{\bitheta}{\boldsymbol{\theta}}\newcommand{\biiota}{\boldsymbol{\iota}}\newcommand{\bikappa}{\boldsymbol{\kappa}}\newcommand{\bilambda}{\boldsymbol{\lambda}}\newcommand{\bimu}{\boldsymbol{\mu}}\newcommand{\binu}{\boldsymbol{\nu}}\newcommand{\bixi}{\boldsymbol{\xi}}\newcommand{\biomicron}{\boldsymbol{\micron}}\newcommand{\bipi}{\boldsymbol{\pi}}\newcommand{\birho}{\boldsymbol{\rho}}\newcommand{\bisigma}{\boldsymbol{\sigma}}\newcommand{\bitau}{\boldsymbol{\tau}}\newcommand{\biupsilon}{\boldsymbol{\upsilon}}\newcommand{\biphi}{\boldsymbol{\phi}}\newcommand{\bichi}{\boldsymbol{\chi}}\newcommand{\bipsi}{\boldsymbol{\psi}}\newcommand{\biomega}{\boldsymbol{\omega}}\begin{equation}\tag{11}\eqalign{&{Q_n}\left( q \right) \propto {Q_{n - 1}}\left( q \right)\Psi \left( {{{\it{\Delta}} _n};\theta } \right) {\rm{\ if\ the\ }}n{\rm{th\ trial\ was\ \rm answered\ correctly}} \cr&{Q_n}\left( q \right) \propto {Q_{n - 1}}\left( q \right)\left( {1 - \Psi \left( {{{\it{\Delta}} _n};\theta } \right)} \right) {\rm{\ if\ the\ }}n{\rm{th\ trial\ was\ \rm answered\ incorrectly}} \cr}, \end{equation}

the symbol ∝ meaning “is proportional to,” since the normalization is not important. In this way, our probability density function for log threshold, *Q_n_*(*q*), is updated on successive trials. We began with a flat prior: the distribution *Q*_0_(*q*) was set to the same value for all values of log threshold. Numerically, *Q_n_*(*q*) was evaluated at 1000 equally spaced values from *q*_min_ = 0 to *q*_max_ = 3.56 log_10_ arcsec.

After completion of the test, we used the posterior to estimate the precision with which the threshold has been obtained. We took half the distance between the 84th and 16th percentile of *Q_n_*(*q*) as an estimate of the standard deviation of the sampling distribution of the threshold, that is, the standard error on the threshold estimate.

##### Fixed-Step Staircase

The introductory phase is followed by five trials of a fixed-log-step 1-down, 1-up staircase. Here, disparity is reduced by a factor of 1.4 for each correct answer and increased by a factor of 1.4 for each incorrect answer (capped as described below). This was because if we go straight to the Bayesian staircase described in the following paragraph, the disparity jumps straight to a low value, which pilot work suggested can be disconcerting for inexperienced participants. However, the results obtained during the fixed-step trials were still used to update the probability density function *Q_n_*(*q*) as described above (Equation 11).

##### Bayesian Staircase

After five trials of the fixed-step staircase, the test moved into a standard adaptive Bayesian staircase procedure. Now, the disparity on each trial was selected to be the mean of the probability density function *Q_n_*(*q*), as recommended by King-Smith et al.^27^ The staircase terminated once 20 trials had been completed, not including trials during the initial introductory phase.

##### Disparity Cap

Because our logistic psychometric function is monotonic, the staircase procedure responds to incorrect trials by increasing the disparity. The intention is to make the task easier; the problem is that excessively large disparities eventually become harder to detect as they exceed the range of stereopsis. To avoid this, if the disparity chosen for the next trial by the methods described above exceeded 1200 arcsec, it was capped at 1200 arcsec.

##### Stereoblind Participants

Our staircase procedure aims to adjust the disparity so that the participant is correct on 75% of trials. Estimates of stereoblindness in the non–visually impaired population range from 3% to 14%,^28^–^32^ and the proportion will typically be higher in eye clinics. It is therefore important to ensure that the stereotest does not discourage stereoblind participants. For this reason, if the current threshold estimate exceeded 1200 arcsec, not only was the disparity capped at this value, but also the nonstereo cue used in the initial practice trials was replaced with full opacity. This served two purposes. First, it ensured that stereoblind participants could also perform well on the task. Second, it provided us with “catch trials,” enabling us to distinguish issues with cooperation or understanding due to problems with stereovision; that is, if a participant scores at chance on the stereo trials but perfectly on trials with a nonstereo cue, then we conclude that they are having particular problems detecting the disparity. But if a participant is at chance on the cued trials also, we conclude that they have not understood the task or are not motivated to perform it (e.g., they are a small child who is enjoying tapping the screen at random). ASTEROID thresholds above 1000 arcsec are not meaningful but indicate a participant performing at chance. In the figures, all thresholds above 1000 arcsec were replaced with a notional value of 1000 arcsec.

### 3D TV Stereotest

We implemented our stereotest as closely as possible on a 3D TV (model 47LD 920; LG Electronics, Seoul, Korea) as well as on a tablet. The LG TV was a 47-inch passive stereoscopic display of the row-interleaved patterned-retarder type. The resolution of this display was 1920 × 1080 pixels, or in 3D mode, 1920 × 540 in each eye after the row interleaving. The code in this case was implemented in MATLAB (Psychophysics Toolbox 3; MathWorks, Natick, MA).^33^

### Participants

Participants were a total of 86 adults aged 18 to 79 years, recruited from the Newcastle University Institute of Neuroscience Research Volunteer pool. Different participants completed different experiments, so summaries of participant demographics will be given in the relevant Results sections. All gave informed written consent to participate after explanation of the nature and possible consequences of the study. The research followed the tenets of the Declaration of Helsinki. The studies presented here were approved by the Newcastle University Faculty of Medical Sciences Research Ethics Committee (01078_2/2016); pilot work in the Royal Victoria Eye Clinic was approved by the North East – Tyne & Wear South Research Ethics Committee (15/NE/0330).

### Simulated Limits of Agreement

To obtain the 95% limits of agreement shown in Figure 8, we ran a simulated version of our ASTEROID test with model observers. The model observer's probability of answering correctly on a simulated trial with a given disparity was given by the logistic psychometric function defined in Equation 9, with a particular value of *θ* corresponding to the stereo threshold of the model observer. In general, the parameters of the model observer (their lapse rate *λ*, the steepness of their psychometric function as governed by *b*) could be different from the values *λ* = 0.03, *b* = 4.885/log_10_ arcsec assumed by ASTEROID; values are specified in the Results section.

**Figure 7:**
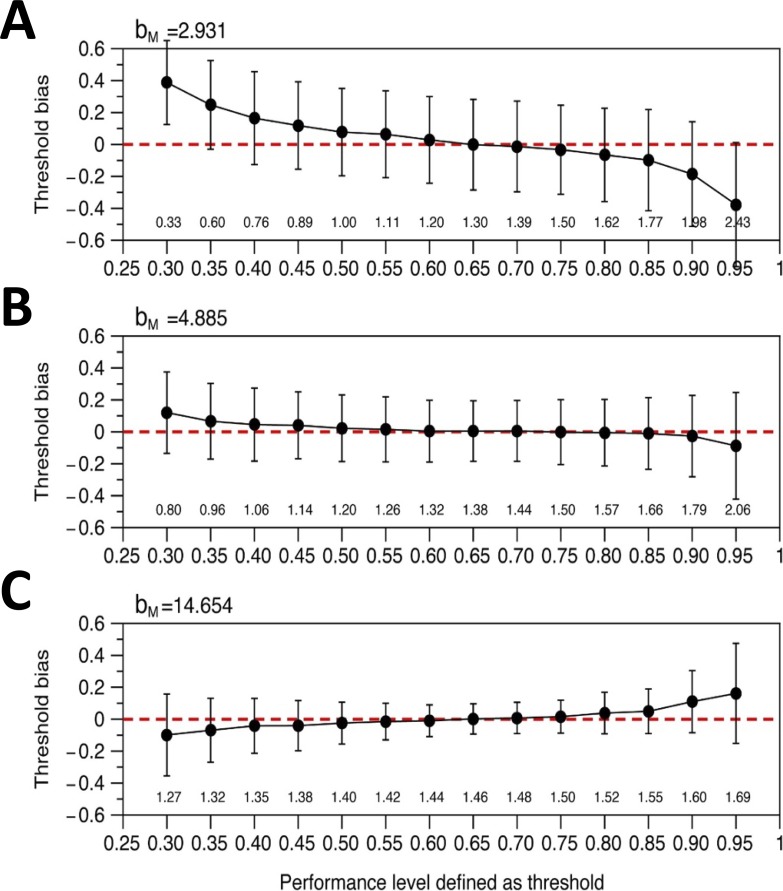
How accuracy and precision of threshold estimates depends on the level defined as threshold performance. The horizontal axis shows Θ, the proportion of correct answers defined as “threshold” (see Equation 8) and (above) the corresponding disparity values in log_10_ units. The vertical axis on each plot shows the bias in estimated threshold, that is, the difference between the threshold estimate returned after 20 trials and the model's true threshold θ_m_, expressed in log_10_ stereo threshold units. By definition, Ψ(θ_m_) = Θ. Black dots show the mean threshold estimate of 2000 simulated staircase procedures, as described in the Methods. Error bars show the standard deviation. The bias is close to zero, and the standard deviation is roughly constant, over a wide range of Θ (∼50%–80%). The staircase assumes the psychometric function given by Equation 9 with b = 4.885/log_10_ arcsec, λ = 0.03, g = 0.25, and Θ specified by the horizontal axis. The model observer is assumed to have a psychometric function with the same form and the same values of λ and g, but different slope parameters b_M_, as specified in each panel.

**Figure 8:**
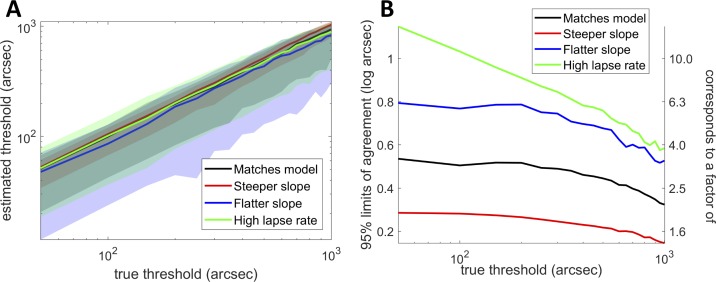
(A) Threshold estimates obtained on a simulated version of ASTEROID for four different example model observers. (B) Ninety-five percent limits of agreement for pairs of threshold estimates from the same four model observers. Model observers differ in their true stereo threshold, indicated by position on the horizontal axis, and in their lapse rate and the steepness of their psychometric function, as indicated by the different colored curves. Black: λ_M_ = 0.03, b_M_ = 4.885/log_10_ arcsec; red: λ = 0.03, b_M_ = 14.654/log_10_ arcsec; blue: λ_M_ = 0.03, b_M_ = 2.931/log_10_ arcsec; green: λ_M_ = 0.1, b_M_ = 4.885. In every case the simulated ASTEROID assumed λ = 0.03, b = 4.885.

We ran a simulated version of ASTEROID with 20 stereo trials and obtained an estimate for the model observer's stereo threshold. We did this 5000 times for each model observer. Because of the probabilistic nature of the psychometric function, different answers were obtained on successive runs. We then looked at all 5000 × 4999/2 pairs of estimates for a given observer and computed the difference in log_10_ stereo threshold. The Bland-Altman 95% limits of agreement are defined as the 95% confidence interval on this difference. Since the differences are close to normally distributed, this spans ±1.96 times the standard deviation of the differences, *s*. That is, one can be 95% sure that the absolute difference between two log threshold estimates will be smaller than 1.96 *s*. This corresponds to a factor of 91 × 10*^s^* in the stereo thresholds themselves.

### Correcting Threshold for Tasks With Different Numbers of Alternatives

In this paper, we wish to compare thresholds obtained from ASTEROID with thresholds on a 2AFC task from a previous study.^9^ Both defined *threshold* as the disparity needed to reach a performance of 75% correct. The problem is that for a 4AFC task, more signal is required to reach 75% than for a 2AFC task. The 2AFC thresholds will therefore be lower, even if everything else is identical. To quantify this, we rearrange Equation 8 into

\newcommand{\bialpha}{\boldsymbol{\alpha}}\newcommand{\bibeta}{\boldsymbol{\beta}}\newcommand{\bigamma}{\boldsymbol{\gamma}}\newcommand{\bidelta}{\boldsymbol{\delta}}\newcommand{\bivarepsilon}{\boldsymbol{\varepsilon}}\newcommand{\bizeta}{\boldsymbol{\zeta}}\newcommand{\bieta}{\boldsymbol{\eta}}\newcommand{\bitheta}{\boldsymbol{\theta}}\newcommand{\biiota}{\boldsymbol{\iota}}\newcommand{\bikappa}{\boldsymbol{\kappa}}\newcommand{\bilambda}{\boldsymbol{\lambda}}\newcommand{\bimu}{\boldsymbol{\mu}}\newcommand{\binu}{\boldsymbol{\nu}}\newcommand{\bixi}{\boldsymbol{\xi}}\newcommand{\biomicron}{\boldsymbol{\micron}}\newcommand{\bipi}{\boldsymbol{\pi}}\newcommand{\birho}{\boldsymbol{\rho}}\newcommand{\bisigma}{\boldsymbol{\sigma}}\newcommand{\bitau}{\boldsymbol{\tau}}\newcommand{\biupsilon}{\boldsymbol{\upsilon}}\newcommand{\biphi}{\boldsymbol{\phi}}\newcommand{\bichi}{\boldsymbol{\chi}}\newcommand{\bipsi}{\boldsymbol{\psi}}\newcommand{\biomega}{\boldsymbol{\omega}}\begin{equation}\tag{12}{\log _{10}}\theta = A - {1 \over b}\ln {{\left( {1 - \lambda - {\rm{\Theta }}} \right)} \over {\left( {{\rm{\Theta }} - g} \right)}}.\end{equation}

We can then work out the ratio between *θ*_2_, obtained with *g*_2_ = 0.5, and *θ*_4_, obtained with *g*_4_ = 0.25, for the same value of Θ = 75%. We assume that *A* and *b* remain the same in both cases. This assumption differs from a common way of modeling *m*-alternatives in the literature.^34^,^35^ That model assumes that an ideal observer selects the target if and only if the noisy signal, drawn from *ϕ*(*x* − *d*′), exceeds all (*m* − 1) samples drawn from noise distributions *ϕ*(*x*). This model also generates a psychometric function that rises from *g* = 1/*m* to optimal, but the slope of the psychometric function is nonzero at *d*′ = 0. This is because with no internal threshold even vanishingly small amounts of signal help push performance above chance. In contrast, our model assumes that all subthreshold signals are equivalent and undetectable. This implies a psychometric function that is initially flat before rising from *g* = 1/*m* to optimal, which accords better with the psychometric functions actually observed, at least on a disparity-detection task. Additionally, the threshold-free model assumes that observers compare all *m* values and pick the largest, whereas our model assumes that the target is detected if and only if the noisy signal exceeds the internal threshold, without the need for comparison. This better agrees with empirical evidence that the time taken to complete a trial grows only slowly with the number of alternatives, as if the target usually “pops out.”^17^ While these details are not critical to our argument, this explains why we assume here that *A* and *b* in Equation 8 are independent of the number of alternatives, which would not be the case in a model without a threshold.

However, the value of *λ* does change. Recall that *λ* is the probability of answering incorrectly even for a stimulus well above threshold. If we assume a fixed probability λ^*^ of ignoring the signal, then *λ* = λ^*^(1 − *g*).

Using Equation 12, we find that

$$\newcommand{\bialpha}{\boldsymbol{\alpha}}\newcommand{\bibeta}{\boldsymbol{\beta}}\newcommand{\bigamma}{\boldsymbol{\gamma}}\newcommand{\bidelta}{\boldsymbol{\delta}}\newcommand{\bivarepsilon}{\boldsymbol{\varepsilon}}\newcommand{\bizeta}{\boldsymbol{\zeta}}\newcommand{\bieta}{\boldsymbol{\eta}}\newcommand{\bitheta}{\boldsymbol{\theta}}\newcommand{\biiota}{\boldsymbol{\iota}}\newcommand{\bikappa}{\boldsymbol{\kappa}}\newcommand{\bilambda}{\boldsymbol{\lambda}}\newcommand{\bimu}{\boldsymbol{\mu}}\newcommand{\binu}{\boldsymbol{\nu}}\newcommand{\bixi}{\boldsymbol{\xi}}\newcommand{\biomicron}{\boldsymbol{\micron}}\newcommand{\bipi}{\boldsymbol{\pi}}\newcommand{\birho}{\boldsymbol{\rho}}\newcommand{\bisigma}{\boldsymbol{\sigma}}\newcommand{\bitau}{\boldsymbol{\tau}}\newcommand{\biupsilon}{\boldsymbol{\upsilon}}\newcommand{\biphi}{\boldsymbol{\phi}}\newcommand{\bichi}{\boldsymbol{\chi}}\newcommand{\bipsi}{\boldsymbol{\psi}}\newcommand{\biomega}{\boldsymbol{\omega}}{\log _{10}}{{\theta _4} \over {\theta _2}} = {1 \over b}\ln \left[ {{{\left( {1 - {\lambda ^*}\left( {1 - {g_2}} \right) - {\rm{\Theta }}} \right)\left( {{\rm{\Theta }} - {g_4}} \right)} \over {\left( {1 - {\lambda ^*}\left( {1 - {g_4}} \right) - {\rm{\Theta }}} \right)\left( {{\rm{\Theta }} - {g_2}} \right)}}} \right]{\rm {.}}$$

We have assumed *λ* = 0.03 for *g*_4_ = 0.25, which implies λ^*^ = 0.04. With Θ = 75% and *b* = 4.885/log_10_ arcsec, the right-hand side of this Equation evaluates to 0.151. That is, with our assumptions about the observer, log thresholds on a 2AFC task should be lower by 0.151 log_10_ arcsec than log thresholds on the equivalent 4AFC task, corresponding to a factor of around 1.4 for thresholds in arcseconds.

## Results

### Simulation 1: Choice of Threshold Performance Level

For a clinical test, it is desirable that test-retest reliability should be as high as possible, but the number of trials available to achieve this is necessarily small. One factor that influences accuracy is the choice of the performance level defined as *threshold*. The sweat factor or sweet point is the stimulus strength that minimizes the variability of the psychometric function for a given number of trials.^36^–^38^ From this stimulus level, we can obtain the performance or proportion of correct responses of the psychometric function. For our 4AFC and the logistic function, this performance is 68% (without lapses) and a little lower, 66%, with *λ* = 0.03 (values estimated by minimizing Equation A1 from Shen and Richards^39^). This is a low performance level for a clinical test aimed at children. For comparison, the Randot stereotest targets performance around 78% correct, whereas the TNO stereotest targets around 85%.^40^ If we chose Θ = 66%, the staircase would be aiming to present disparities where the patient is wrong nearly half the time. We were concerned that this would be demotivating in a clinical test aimed at children. Yet, we were also keen to maximize precision and thus reliability. We therefore carried out simulations to assess the effect of different choices for Θ, the performance level defined as threshold.

The results are shown in Figure 7. The bias (dots) and standard deviation (error bars) of threshold estimates are plotted as a function of Θ for values from 30% to 95% for three different model observers whose psychometric functions have different slope parameters *b*_M_. Unsurprisingly, thresholds are obtained with greatest precision (error bars are lower) for the observer with the highest slope (C: *b*_M_ = 14.654). As a function of Θ, regardless of observer slope, the lowest biases and standard deviations are obtained near Θ = 66%. The standard deviation becomes large for very low or very high Θ, and when the model slope is not as assumed by the staircase (A, C), biases are also possible for these extremes. However, across the range from Θ = 50% to 80%, there is in fact very little difference in the quality of the threshold estimates. We therefore chose a definition of threshold performance level toward the upper end of this range, Θ = 75%.^41^ This is close to threshold performance levels targeted by current stereotests. It means that patients using ASTEROID will find they are correct around three times out of four, even toward the end of the test, helping to prevent discouragement. All subsequent results in this paper are for Θ = 75%.

### Simulation 2: Test-Retest Reliability

ASTEROID is intended as a clinical stereotest. Involvement from clinicians strongly indicated that to be useful, the test must above all be quick.^42^ Therefore, in a clinical context, thresholds must be estimated from very few trials. ASTEROID uses four practice trials, with a nonstereo cue, and stereo thresholds are estimated from the subsequent 20 stereo trials. This small number of trials strongly limits the test-retest reliability achievable.

Figure 8A shows the distribution of values obtained from simulated model observers after 20 trials. The horizontal axis shows the true threshold of the model observer. The solid lines show the median estimated threshold, while the shaded region marks the 2.5% and 97.5% percentiles, based on 5000 tests. The different colors represent different model observers.

Figure 8B shows the Bland-Altman 95% limits of agreement^43^,^44^ for the same model observers. This is defined such that one can be 95% confident that the absolute difference between two observations will be less than the 95% limit of agreement (see Methods). This means that if the first observation is *q*, then the second could be as high as *q* + limit of agreement or as low as *q* − limit of agreement. For stereoacuity expressed in log arcseconds, the limit of agreement has units log_10_ arcseconds. If we express stereoacuity in arcseconds, the limit of agreement becomes a factor,^44^ as shown on the right-hand vertical axis. Limits of agreement are slightly smaller for observers with larger thresholds, reflecting the fact that ASTEROID will not present disparities larger than 1200 arcsec (see Methods, Disparity Cap). The black line shows results for an observer matching the ASTEROID assumptions; that is, their lapse rate *λ*_M_ = 0.03 and slope parameter *b*_M_ = 4.885/log_10_ arcsec. Better reliability, that is, tighter limits, are obtained for the model observer shown in red, who has a steeper psychometric function, *b*_M_ = 14.654/log_10_ arcsec. This is because a threshold is more tightly defined when the psychometric function rises more steeply from chance to optimal, as we saw in Figure 7. Conversely, reliability is worse for the model observer shown in blue, where *b*_M_ = 2.931/log_10_ arcsec. The green curve shows an observer with *b*_M_ = 4.885/log_10_ arcsec, but a high lapse rate of 0.1. This might describe a small child who is regularly distracted. Here, limits of agreement are unsurprisingly higher.

For realistic observers, the limit of agreement is around 0.5 log_10_ arcsec or a factor of 3 (∼10^0.5^). That is, with only 20 trials, a second threshold estimate may be a factor of 3 higher or lower than the first threshold estimate, simply due to the stochastic nature of psychophysical judgments. This places a fundamental limit on the test-retest reliability we can expect from ASTEROID.

### Experiment 1: Effect of Viewing Distance

**Figure 9:**
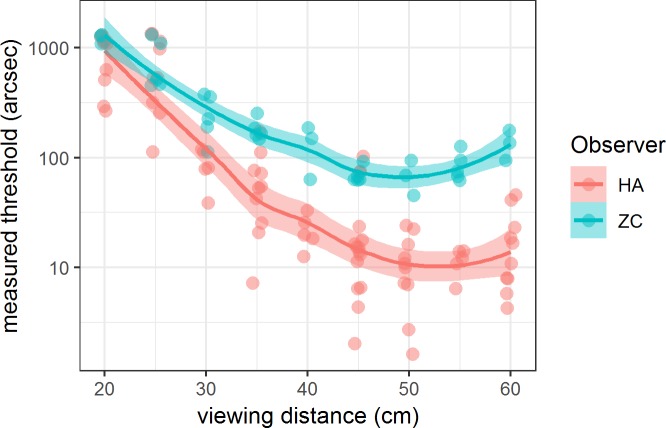
Measured thresholds for two observers as a function of viewing distance. Symbols show measured thresholds (each from at least 20 nonpractice trials), slightly jittered to avoid overlap. Lines show trend obtained by loess smoothing; ribbon shows 95% confidence interval.

The Commander 3D autostereo technology does not work equally well at all viewing distances. When it is held too close, there may be substantial cross talk, and/or this may vary across the screen. The effect of this is to increase stereo thresholds or even to make thresholds unobtainable at short distances. Figure 9 quantifies this for two observers, authors HA and ZC, both females aged 17 with normal vision, who took a total of 116 threshold measurements while viewing the tablet from nine different distances.

Clearly, the threshold estimates depend strongly on viewing distance, falling steeply as distance increases from 25 to approximately 35 cm. This is not due to failures in the correction for viewing distance. First, the observers wore the sticker, so the tablet was able to estimate their viewing distance and correct the parallax accordingly, and we confirmed it obtained largely accurate and certainly bias-free distance estimates. Second, a given parallax corresponds to larger disparities at closer viewing distances. According to Equation 4, a parallax of 1 pixel is 188 arcsec at 25 cm but 78 arcsec at 60 cm, corresponding to a factor of 2.4 over the range of distances used in Figure 5. Thus, a failure to correct for viewing distance would tend to produce erroneously low threshold estimates at close viewing distances and erroneously high thresholds at large viewing distances—the opposite of the pattern observed. The relatively constant thresholds observed for distances over 35 cm confirm that the correction for distance is working. Rather, the steep increase in measured thresholds for close viewing seems to be a property of the device's parallax barrier autostereo. Fortunately, most adults spontaneously hold the device at a viewing distance of at least 35 cm. In subsequent sections, we removed any observers for whom the device reported a mean viewing distance less than 35 cm.

### Experiment 1: Estimating Reliability

We can also use this data to examine the estimate of reliability provided by the staircase procedure itself. Observer ZC collected 24 thresholds at 35 cm or greater; her mean threshold was 2.0 log_10_ arcsec (103 arcsec) and the SD was 0.21 log_10_ arcsec. In each of the individual threshold measurements, the posterior distribution for the log threshold after 20 trials generally resembles a Gaussian. We therefore estimated the standard deviation of each individual threshold as half the distance between the 84% and 16% percentiles for the posterior. The mean SD of the 24 posterior distributions for ZC is 0.17 log_10_ arcsec, with rather little variation (SD of the SD estimates = 0.02 log_10_ arcsec), just slightly larger than the same as the overall standard deviation of the thresholds (0.21 log_10_ arcsec). For observer HA, these numbers were *n* = 55 thresholds; mean = 1.2 log_10_ arcsec (16 arcsec), SD = 0.43 log_10_ arcsec; the standard deviation estimated from staircases was 0.19 ± 0.05 log_10_ arcsec. Thus, observer ZC was nearly as consistent as possible given the staircase; observer HA showed more variability.

### Experiment 2: Test-Retest Reliability with Human Observers

**Figure 10:**
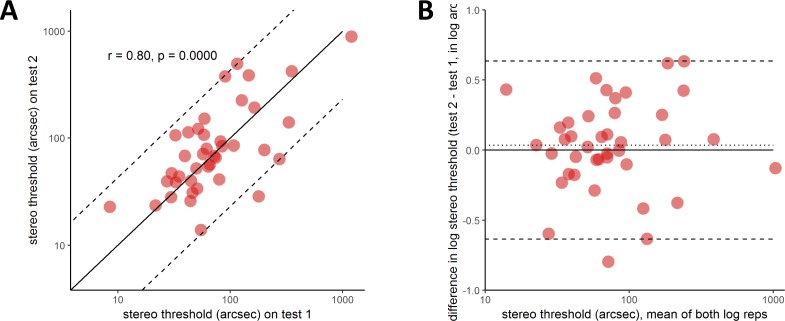
Test-retest reliability of ASTEROID assessed from 40 adult observers. (A) Stereo threshold on second test plotted against stereo threshold on first test. Solid line = identity, dashed line = 1.96 times standard deviation of difference in log_10_ arcsec (95% limits of agreement.) (B) Bland-Altman plot showing test-retest agreement on ASTEROID. Vertical axis shows the difference, in log arcseconds, between the results of two tests. The horizontal axis shows the mean result (the arithmetic mean of the log thresholds, which is the geometric mean of the thresholds in arcseconds). The horizontal dotted line shows the mean of all 35 differences. The horizontal dashed lines show the ±1.96 SD of the differences, which are Bland & Altman's 95% limits of agreement.

To examine reliability in more detail, 40 naive adult observers (21 females, 19 males; age range 19–78 years, but mainly under 30) completed two ASTEROID tests. Figure 10 shows the agreement between their first and second threshold estimates on each test. Figure 10A shows thresholds on the second test plotted against thresholds on the first test. The Pearson correlation coefficient is 0.80 (*P* < 10^−7^) and Spearman coefficient is 0.63 (*P* < 10^−4^). For comparison, Bosten et al.^31^ report a Spearman test-retest correlation coefficient of 0.67 on a laboratory stereotest using 50 trials. Thus, ASTEROID's value of 0.63 for 20 stereo trials stands up well.

Figure 10B shows the same data presented as a Bland-Altman plot.^43^ The vertical axis shows the difference between the results of two tests on the same observer. The horizontal axis shows the mean result. The horizontal dotted line shows the mean of all 40 differences. This is not significantly different from zero (paired *t*-test on log thresholds, *P* = 0.57), meaning that there is no evidence for systematic changes, for example, due to practice or fatigue. The horizontal dashed lines show the Bland-Altman 95% limits of agreement, equal to ±1.96 times the SD of the differences. This is equivalent to the colored lines in Figure 8B, except there we folded the plot and showed only the upper value. The 95% limit of agreement is 0.64, corresponding to a factor of 4.3 (that is, the range is ±0.64 log_10_ arcsec, which corresponds to multiplying or dividing the threshold in arcseconds by a factor of 4.3). This is comparable to the values obtained from our simulations (Fig. 8B), confirming that with real observers the test achieves close to the maximum reliability permitted by the staircase procedure given the short number of trials.

We can again compare this estimate of reliability with the error estimates provided by the staircase procedure. The average standard deviation estimated from the staircase was 0.18 log_10_ arcsec for these 35 participants, very close to the values obtained for observers ZC and HA in the previous section. Observers ZC and HA each performed >20 threshold estimates, so we were able to compare the theoretical standard deviation estimated from the staircase with the empirical standard deviation from multiple measurements in the same observer. In this section, each of the 35 observers carried out only two measurements, which does not enable an accurate measurement of standard deviation in individuals. However, if we assume that all observers have the same standard deviation, we can compare this empirical population standard deviation with the staircase estimate. Assume that each observer has a unique threshold, *q*, and that each time we try and measure this, we get a value drawn from a normal distribution with mean *q* and SD σ (all in log_10_ arcseconds). Then, the standard deviation of the difference between two measurements for the same observer is also drawn from a normal distribution, with mean 0 and SD σ√2. Our staircase estimates that σ = 0.18 log_10_ arcsec, so σ√2 = 0.25 log_10_ arcsec. Empirically, the standard deviation of the difference between two threshold measurements in the same observer (Fig. 10B) was 0.32 log_10_ arcsec, slightly larger but close to the value predicted by the staircase. This provides further confirmation that the statistics observed with human participants are as expected from simulations.

### Experiment 3: Within-Subjects Comparison of ASTEROID With a Stereotest on a 3D TV

**Figure 11:**
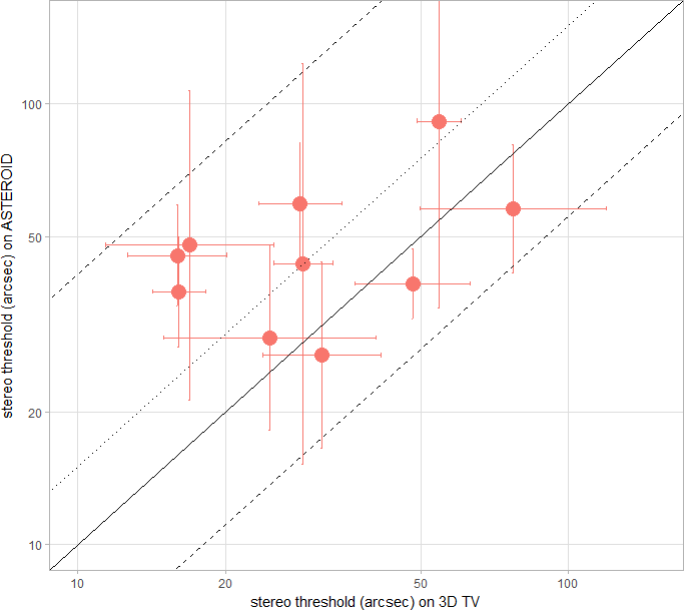
Results from 10 adults who completed three tests with ASTEROID (vertical axis) and three tests with an equivalent test presented on a stereoscopic 3D TV (horizontal axis). Data points show the mean of the three values obtained from each test (mean of log arcsecond) and error bars show ±1 SD. The solid line shows the identity, that is, perfect agreement. The dotted line shows the identity offset by the mean difference between the results, while the dashed lines show the Bland-Altman 95% limits of agreement between the two tests, that is, the mean difference ± 1.96 times the standard deviation of the difference. Means are computed on log thresholds, which correspond to the geometric mean of the thresholds in arcseconds.

Of course, greater reliability can be obtained with more presentations. Ten participants completed three tests with ASTEROID and three tests with a similar program presented on a stereoscopic 3D TV, so there was a total of 60 stereo presentations with each type of equipment. Figure 11 compares results (average of 3 scores) on the two types of equipment. Participants tended to obtain a slightly worse score on ASTEROID (mean difference of 0.18 log arcsec or a factor of 1.5, shown by the dotted line; *P* = 0.03, paired sample *t*-test on log thresholds). The correlation between scores on the two tests was not significant, partly because of the small number of participants and partly because their thresholds were all very similar (all under 100 arcsec).

The dashed lines show the Bland-Altman 95% limits of agreement. These were ±0.22 log_10_ arcsec or a factor of 1.7. This is for a comparison between two different tests, so the test-retest agreement between ASTEROID must be at least as good. Thus, while we found previously that the limits of agreement corresponded to a factor of 4.6 for a single ASTEROID test (20 presentations), this is reduced to 1.7 if one takes the geometric mean of three ASTEROID thresholds (60 presentations).

### Experiment 4: Between-Subjects Comparison of ASTEROID With a Stereotest on a 3D TV

**Figure 12:**
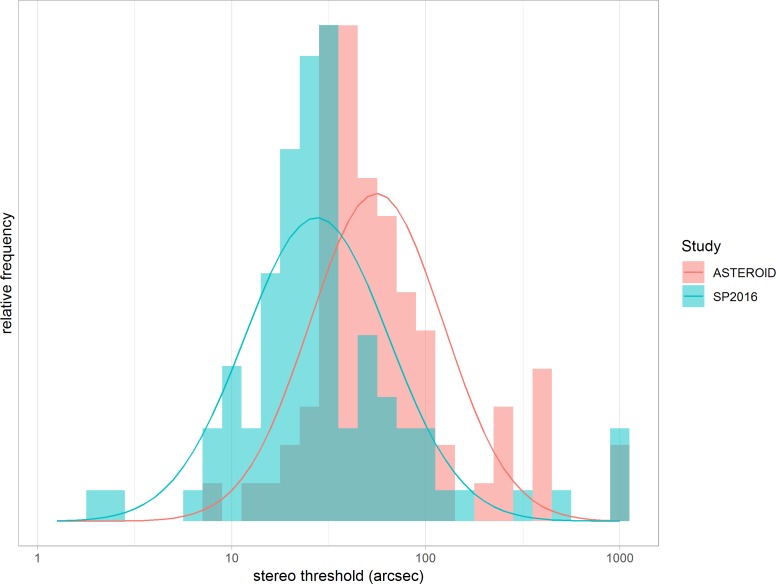
Distributions of stereo thresholds obtained on ASTEROID (red, 74 participants) and from a similar test presented on a stereoscopic 3D TV in an earlier study (blue,^9^ 91 participants, corrected from 2AFC to 4AFC as described in the text). In both cases participants were adults aged from 18 to nearly 80 years. The thin lines show Gaussian distributions with the same mean and standard deviation as the corresponding data-set. Means and standard deviations excluded threshold estimates over 1000 arcsec, reflecting the view that stereoacuity reflects a mixture of two distributions: a roughly log-normal distribution (shown with the curves here) and a smaller proportion of observers who are stereoblind.^45^ Threshold estimates over 1000 arcsec were considered stereoblind and are plotted at 1000 arcsec.

We also compared the stereo thresholds measured for adults using ASTEROID with a large data-set from a previous study.^9^ The stereotest used in the previous study was similar to ASTEROID but had two major differences: (1) it was presented on the same stereoscopic 3D TV, viewed at 200 cm, instead of on an autostereo tablet, and (2) it used a Bayesian staircase with 35 trials instead of 20. Figure 12 compares the distribution of stereo thresholds from 91 adult participants in that study (SP2016, blue) with that from a different group of 74 adult participants tested on ASTEROID (red). Participant demographics were SP2016: *n* = 91, age range 18 to 73 years, mean 31, SD 17; ASTEROID: *n* = 74; 49 female, 25 male; age range 18 to 79 years, mean 26, SD 12.

A subtlety is that Serrano-Pedraza et al.^9^ used a two-alternative version of the task but still defined threshold as a performance of 75% correct. On a two-alternative task, chance is 50% correct, so less signal is required to reach 75% correct. We showed in the Methods (Correcting Threshold for Tasks with Different Numbers of Alternatives) that this means 2AFC thresholds should be lower by a factor of around 1.4: an observer who scored 100 arcsec in SP2016 should score 140 arcsec on ASTEROID. We therefore multiplied all the SP2016 thresholds by 1.4 before plotting them in Figure 12.

In both cases, the distribution of stereo thresholds for non-stereoblind observers is roughly normal on log axes, and the standard deviations are very similar for the two studies. Excluding participants who scored over 1000 arcsec, the standard deviation is 0.37 log_10_ arcsec for SP2016 and 0.34 log_10_ arcsec for ASTEROID, corresponding to a factor of 2.2 (that is, a range of ±1 SD corresponds to multiplying or dividing the threshold in arcseconds by a factor of 2.2). The fact that the between-subjects standard deviations of both samples is so similar, and in fact slightly smaller for ASTEROID, also implies that the within-subject “noise” on the measurement must be similar between the two tests, that is, the decrease from 35 to 20 presentations has not impacted reliability substantially.

The means with the two tests, however, differ significantly (*p* < 10^−6^, *t* = −5.4, Welch two-sample *t*-test on log thresholds below 1000 arcsec), even after the correction for 2AFC versus 4AFC. In SP2016, the mean was 1.44 log_10_ arcsec, corresponding to 27 arcsec (median 26 arcsec), whereas on ASTEROID it was 1.75 log_10_ arcsec, corresponding to 57 arcsec (median 47 arcsec). That is, stereo thresholds were around twice as high for the sample tested on the 3D tablet as compared to a different sample tested on the stereoscopic 3D TV.

### Experiment 5: Comparing ASTEROID Results With the Randot Preschool Stereotest

**Figure 13:**
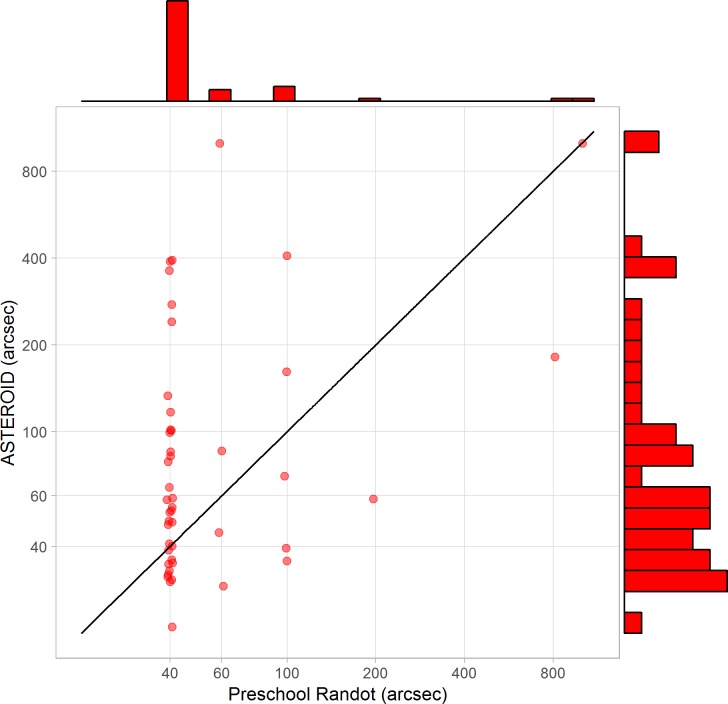
Results from 52 adults who completed one test with ASTEROID (vertical axis) and one with the Randot Preschool stereotest (horizontal axis). Points have been jittered slightly horizontally to avoid symbol overlap. The black line marks the identity. Marginal distributions are shown along the sides. The correlation coefficient between log thresholds is r = 0.37 (P = 0.01).

Forty-six adults had their stereoacuity tested with ASTEROID and also with Randot Preschool stereotest (one test in each case). Participant demographics were 29 female, 17 male, age range 18 to 79 years, mean 30, SD 14. We used the commercially available three-book version of the Randot Preschool stereotest from the Stereo Optical Company (Chicago, IL), administered at 40 cm. This test permits only scores of 40, 60, 100, 200, 400, and 800 arcsec, indicated by the gridlines in Figure 13. In contrast, ASTEROID can return a score anywhere between 1 and 1200 arcsec, although values >1000 arcsec are considered stereoblind. In Figure 13, participants who could not complete the 800 arcsec level of the Randot Preschool stereotest, or who obtained an ASTEROID score above 1000 arcsec, were assigned a notional value of 1000 arcsec on that test.

As in Figure 12, the distribution of non-stereoblind thresholds on ASTEROID is roughly normal on log axes. In contrast, three-quarters (34 out of 46) of our participants obtained the best possible score on the Randot Preschool stereotest: 40 arcsec. Despite the concentration of Randot Preschool scores at 40 arcsec, stereo thresholds on the two tests are correlated (*r* = 0.37, *P* = 0.01, Pearson product-moment correlation on log thresholds). The mean score for non-stereoblind participants on ASTEROID is nearly twice as high, 70 arcsec on ASTEROID compared to 51 for Randot Preschool stereotest (mean of log thresholds), a ratio of 1.38. This is similar to the ratio estimated in experiment 4 comparing ASTEROID to the 3D TV test.

Previous laboratory measurements of stereoacuity have also obtained a roughly log-normal distribution of stereoacuity,^9^,^16^,^31^,^46^ with standard deviation variously reported as 0.23 log_10_ arcsec^46^ or 0.37 log_10_ arcsec^9^ for non-stereoblind observers. The standard deviation for non-stereoblind observers with ASTEROID in Figure 13 is 0.35 log_10_ arcsec, in line with these estimates. Thus, it is likely ASTEROID has correctly captured the variation within our sample of observers. In the Randot Preschool stereotest, most of this variation is collapsed to a single score of 40 arcsec, obscuring genuine differences in stereoacuity between observers.

## Discussion

In this paper, we have described ASTEROID, a new stereotest presented on an autostereo 3D tablet computer and designed for clinical use. We have presented preliminary results addressing the distributions of stereo thresholds obtained with adults on ASTEROID and its test-retest reliability. We have compared it to a similar test administered on a 3D monitor with controlled viewing distance and passive polarizing 3D glasses and also to a widely used clinical stereotest, the Randot Preschool stereotest. In Table 3, we summarize how ASTEROID compares to current near clinical stereotests.

**Table 2:** Symbols Used in This Paper

Symbol	Value in ASTEROID Test (where relevant)	Meaning
*I*		Viewer's interpupillary distance, in centimeters; Figure 2.
*V*		Viewing distance from the participant's eyes to the tablet screen, in centimeters; Figure 2.
*p*		Size of a physical pixel on the tablet screen, in centimeters. The effective size of an H-pixel^4^ is 2*p*.
*P*		Screen parallax, in centimeters; Equation 4, Figure 2.
*D*_H_		Screen parallax, in pixels of the half-images (H-pixels), Figure 2.
*D*_I_		Screen parallax, in physical pixels of the image on the screen (I-pixels); Equation 1, Figure 2.
*d*		Geometrical distance from the viewer to a virtual object, as implied by the disparity; Equation 3, Figure 2.
Δ		Relative disparity between the target and the background, in arcseconds; Equation 4.
*q*	*q* = log_10_(*θ*)	Participant's stereo log threshold, in log_10_ arcseconds.
*Q_n_(q)*		Probability density function for the estimated log threshold after *n* trials; Equation 11.
*θ*	*θ* = 10*^q^*	Participant's stereo threshold, in arcseconds; Equation 9.
Θ	75%	Level of performance defined as “threshold”; Equation 8, Equation 9.
Ψ(Δ;*θ*)		Participant's psychometric function, that is, the probability that they correctly select the target given that the stimulus disparity is Δ and their threshold is *θ*; Equation 9.
*λ*	0.03	Participant's lapse rate; Equation 9.
*g*	0.25	Probability of answering correctly by chance; Equation 9.
*b*	4.885/log_10_ arcsec	Slope parameter of psychometric function; Equation 6, Equation 8, Equation 9.
Δ_max_	1200 arcsec	Largest disparity ever displayed.
*q*_min_	0 log_10_ arcsec = 1 arcsec	Thresholds smaller than *q*_min_ are assigned zero probability.
*q*_max_	3.56 log_10_ arcsec = 3600 arcsec = 1°	Thresholds larger than *q*_max_ are assigned zero probability.

**Table 3:** Norms and Reliability for ASTEROID and for Some Current Near Clinical Stereotests

	Mean Threshold in Non-Stereoblind Adults, arcsec	Population SD, log_10_ arcsec	Bland-Altman 95% Limits of Agreement in log_10_ arcsec = 1.96 SD of Differences Between Test and Retest	Spearman Correlation Between Test and Retest
ASTEROID	57	0.35	±0.64 (raw)	0.63
			±0.56 (quantized)	
TNO	52^50^	0.21^50^	±0.45^50^	0.57^31^
	60^31^		±0.48^31^	
Randot Preschool	51	0.24	±0.59^44^	
Near Frisby	21^50^	0.06^50^	±0.24^44^	
			±0.27^50^	
Randot	29^50^	0.15^50^	±0.34^50^	
Titmus	41^50^	0.05^50^	±0.13^50^	

Values reported without references are from the present study. The “quantized” Limits of Agreement reported for ASTEROID is after mapping the original thresholds to the closest available Randot Preschool stereotest score. This is essential for a fair comparison, since test-retest differences are naturally smaller where only a few discrete scores are available. Antona et al.^50^ reported values only in arcseconds. To convert to log arcseconds, we divided the range (standard deviation or limits of agreement) reported in arcseconds by the population mean threshold in arcseconds and by the natural logarithm of 10. For the means, we have reported the value in arcseconds corresponding to the mean of log_10_ arcsecond values. Antona et al.^50^ reported only the mean of the arcsecond, so we have used those values. Bosten et al.^31^ reported mean and median in arcseconds; we have taken the median of the values in arcseconds in the expectation that this will be closer to the mean of the log values.

Simulations indicate that the major source of error in the ASTEROID thresholds is the small number of trials used (20). The 95% limits of reliability represent a factor of between 2 and 4, depending on the observer characteristics. That is, two thresholds obtained from the same model observer may differ by up to a factor of 2 or 4 simply due to the stochastic nature of perception around threshold. This uncertainty dwarfs other sources of error, such as inaccuracies in the viewing distance measure and in subpixel disparities. With adult human observers, we found that the 95% limits of reliability were 0.64 log_10_ arcsec, corresponding to a factor of 4.3. This includes other sources of error, plus variations in the observer's state (concentration, etc.) as well as stochastics. However, the fact that the number agrees so well with the simulations indicates that stochastics are the major contributor.

**Figure 14:**
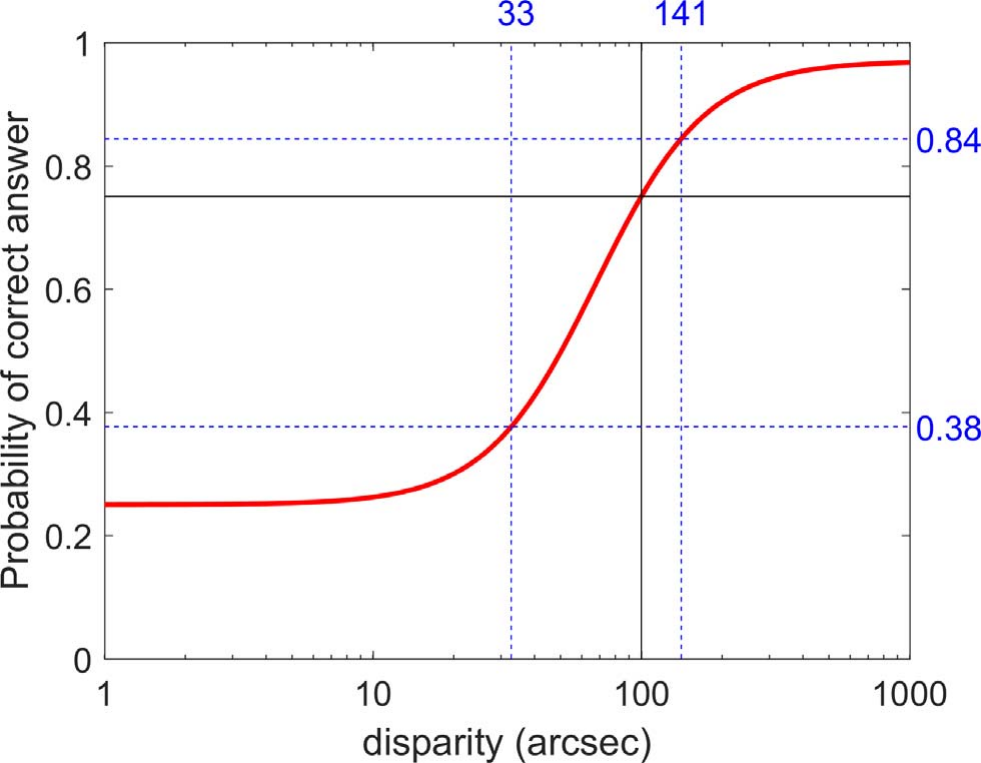
Because disparity psychometric functions are quite shallow, even the steepest part of the curve spans a wide range of disparities. A 4.3-fold change in disparity (e.g., going from 33 to 141 arcsec for a threshold of 100 arcsec) only changes performance from 38% correct to 84% correct. Psychometric function (red curve) is given by Equation 9 with the parameters specified in that section, a threshold of θ = 100 arcsec, and disparity Δ indicated on the horizontal axis.

Ultimately, the precision of the test is limited by the slope of the psychometric function. We assumed a slope parameter *b* of 4.885/log_10_ arcsec (see Equation 9). This means that a 4.3-fold change in disparity around threshold changes performance from 37% to 85% correct (Fig. 14). Thus, in 20 presentations ASTEROID is able to find the disparity where performance is in this range, but more presentations would be required to narrow this down further.

This figure for test-retest reliability is higher than previous estimates for clinical stereotests (Table 3). Adams et al.^44^ report that the 95% limits of agreement with adults are 0.57 log arcsec for the Randot Preschool stereotest, corresponding to a factor of 3.7. However, these figures cannot be directly compared, since current tests return only discrete scores. Successive scores of 80 and 140 arcsec on ASTEROID would be classed as different, whereas on the Randot Preschool stereotest these would both be 100 arcsec and thus agree perfectly. If we quantize ASTEROID threshold estimates to the closest (in log space) available score on the Randot Preschool stereotest and then repeat the Bland-Altman analysis of Figure 10, we find that the 95% limits of agreement for ASTEROID are now 0.56 log_10_ arcsec or a factor of 3.6, almost exactly the same as Adams et al.^44^ found for the Randot Preschool stereotest. Ma et al.^12^ reported high test-retest reliability for their computerized distance stereoacuity test: 95% limits of agreement 0.29 log_10_ arcsec, or a factor of 1.9. However, this is because their participants were drawn from an eye clinic and many were strabismic, so very few could perform the test at all: of the 81 participants, 79 scored “nil” (stereoblind) on the first test and 69 on the retest. In adults with normal binocular vision, this group reports the 95% limits of agreement as 0.47 log arcsec or a factor of 3.0.^13^ From their staircase statistics, Hess et al.^16^ estimate 90% limits of agreement as a factor of 1.9 (they use *Z*-score boundaries of 1.65, not 1.96 as for the 95% limit). However, this tight predicted agreement is not borne out empirically. In their Bland-Altman plot, the 95% limits of agreement are 0.75 log_10_ arcsec or a factor of 5.6. This is a little higher than ASTEROID, even though each of their measurements represents twice as many trials (40–60 trials, compared to 20 for ASTEROID). The greater number of trials required to achieve similar reliability probably reflects their two-alternative task, since each presentation of a two-alternative task conveys half as much information as for a four-alternative task. Like us, Hess et al.^12^ find a 0.79 correlation between two tests on the same observer (Fig. 9A).

To summarize, a single ASTEROID test achieves the same reliability as a single Randot Preschool stereotest, but—as shown in Figure 13—provides more nuanced information about stereoacuity. If higher reliability is required, we saw that taking the mean of 3 ASTEROID log thresholds brings the reliability to a factor of around 1.7.

Thresholds measured with ASTEROID are a little higher than on other tests: on average about 1.5 times higher than those measured with a similar stereotest on an stereoscopic 3D TV (Fig. 10), and around twice as high as thresholds reported by Serrano-Pedraza et al.^9^ (Fig. 11) or obtained with the Randot Preschool stereotest (Fig. 12). Hess et al.^12^ report a modal non-stereoblind threshold of around 1.4 log_10_ arcsec or 25 arcsec, again about a factor of 2 lower than ASTEROID. The difference with Randot Preschool stereotest is not surprising given the very different stimuli. The Randot Preschool stereotest is static and potentially contains monocular cues if participants tilt their head.^4^

The difference with the other tests is more surprising, but presumably relates to differences in the stimuli (e.g., dot size, static versus dynamic dots) and/or display technology. ASTEROID uses relatively large dots: 18 physical pixels across, representing 18 arcmin when the tablet is held at 40 cm. The boundaries of the target thus appear slightly ragged, for the reasons explained in the Methods (Applying Disparity). The dot size is large compared to other tests, for example, 9 arcmin in Serrano-Pedraza et al.^9^ Although the large dots do not impose a Nyquist limit on spatial stereoresolution,^47^ since the stimulus is dynamic, we consider it plausible that they could contribute to higher thresholds. These reasons for differences compared with other tests are not concerning. Clinically, stereo thresholds need to be compared to population norms or to thresholds obtained from the same patient with the same test at a different time, for example before and after amblyopia treatment. Thus, differences due to stimulus properties are not important provided that the test norms are known.

More concerning possible reasons for higher thresholds include cross talk between the left and right images, which is known to impair stereo depth perception.^48^,^49^ As discussed in experiment 1, cross talk is severe when the device is held too close. Even at appropriate viewing distances, cross talk can still occur if the observer tilts the device to left or right. Thus, with the tablet, we depended on participants to hold the tablet correctly so as to eliminate cross talk, whereas in Serrano-Pedraza et al.,^9^ adult participants used a headrest to ensure that their viewing position was correct. Other differences include the pixel resolution of the Commander 3D tablet used for ASTEROID. The minimum relative disparity depictable in whole pixels on the tablet is 118 arcsec at a viewing distance of 40 cm compared to 54 arcsec in Serrano-Pedraza et al.,^9^ yet the mean threshold of adult participants on ASTEROID was 57 arcsec (Fig. 12). Thus, threshold estimates are dependent upon our techniques for obtaining subpixel disparities. Inaccuracies in our measurement of viewing distance could also contribute. If the actual viewing distance was larger than that recorded by the device, then a given screen parallax would be recorded as an erroneously high retinal disparity, leading to an erroneously high estimate of the participant's stereo threshold. These reasons would be unsatisfactory because they could introduce sources of variability. However, some reassurance is provided by the fact that the error estimate from simulations generally aligns well with the variability observed over repeated measurements in the same observer. The main source of variability in ASTEROID thresholds is simply that imposed by the statistics of 20 trials.

An advantage of ASTEROID is that incorrect usage (e.g., tilting the device or holding it too close) tends to produce erroneously high thresholds. For all current clinical tests, incorrect usage can produce erroneously low thresholds, for example, holding the test too close to make the parallax more visible or moving or rotating the test to introduce cross talk or motion parallax. ASTEROID is also easy and intuitive to use and does not require assistance from a test administrator. This makes it potentially feasible for patients to obtain their own ASTEROID thresholds without supervision from a clinician, for example in the waiting room. The result can safely be regarded as an upper bound on the patient's stereoacuity; incorrect usage could produce artifactually poor thresholds, but not artifactually good ones.

## Conclusion

The ASTEROID stereotest makes it possible to carry out a laboratory-standard psychophysical measurement of stereoacuity quickly and easily on a handheld tablet, without the need for 3D glasses. Its test-retest reliability compares well with other clinical stereotests, given that results are not constrained to a set of discrete levels. Whereas all current clinical stereotests offer some form of monocular cue, ASTEROID does not, and it is not possible to obtain erroneously low thresholds by exploiting an artifact. However, observers must view ASTEROID from at least 35 cm and straight on (i.e., with the screen normal lying in their midsagittal plane), to avoid erroneously high thresholds due to cross talk. ASTEROID gives the same log-normal distribution of stereo thresholds as laboratory measures of stereoacuity, but stereo thresholds are approximately twice as high as those measured on a passive stereoscopic 3D monitor. ASTEROID should be especially useful to clinical researchers who need an easy, accurate measurement of stereoacuity.
